# Interactions of Natural Flavones with Iron Are Affected by 7-*O*-Glycosylation, but Not by Additional 6″-*O-*Acylation

**DOI:** 10.1021/acsfoodscitech.3c00112

**Published:** 2023-05-02

**Authors:** Judith Bijlsma, Wouter J. C. de Bruijn, Jamie Koppelaar, Mark G. Sanders, Krassimir P. Velikov, Jean-Paul Vincken

**Affiliations:** † Laboratory of Food Chemistry, Wageningen University & Research, Bornse Weilanden 9, P.O. Box 17, 6700 AA Wageningen, The Netherlands; ‡ Unilever Innovation Centre, Wageningen B.V. Bronland 14, 6708 WH Wageningen, The Netherlands; § Institute of Physics, University of Amsterdam, Science Park 904, 1098 XH Amsterdam, The Netherlands; ∥ Soft Condensed Matter, Debye Institute for Nanomaterials Science, Utrecht University, Princetonplein 5, 3584 CC Utrecht, The Netherlands

**Keywords:** Apium graveolens, apiin, glycosylated flavonoids, polyphenol, ferrous, metal chelation, ligand-to-metal charge transfer, 7-*O*-glycosides, oxidation, complexation

## Abstract

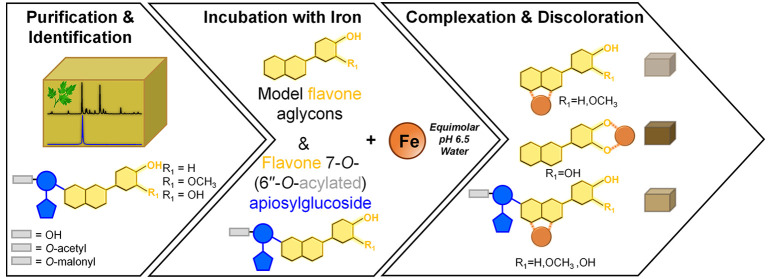

In iron-fortified bouillon, reactivity of the iron ion with (acylated) flavone glycosides from herbs can affect product color and bioavailability of iron. This study investigates the influence of 7-*O*-glycosylation and additional 6″-*O*-acetylation or 6″-*O*-malonylation of flavones on their interaction with iron. Nine (6″-*O*-acylated) flavone 7-*O*-apiosylglucosides were purified from celery (*Apium graveolens*), and their structures were elucidated by mass spectrometry (MS) and nuclear magnetic resonance (NMR). In the presence of iron, a bathochromic shift and darker color were observed for the 7-*O*-apiosylglucosides compared to the aglycon of flavones that only possess the 4–5 site. Thus, the ability of iron to coordinate to the flavone 4–5 site is increased by 7-*O*-glycosylation. For flavones with an additional 3′–4′ site, less discoloration was observed for the 7-*O*-apiosylglucoside compared to the aglycon. Additional 6″-*O*-acylation did not affect the color. These findings indicate that model systems used to study discoloration in iron-fortified foods should also comprise (acylated) glycosides of flavonoids.

## 1 Introduction

**Figure 1:**
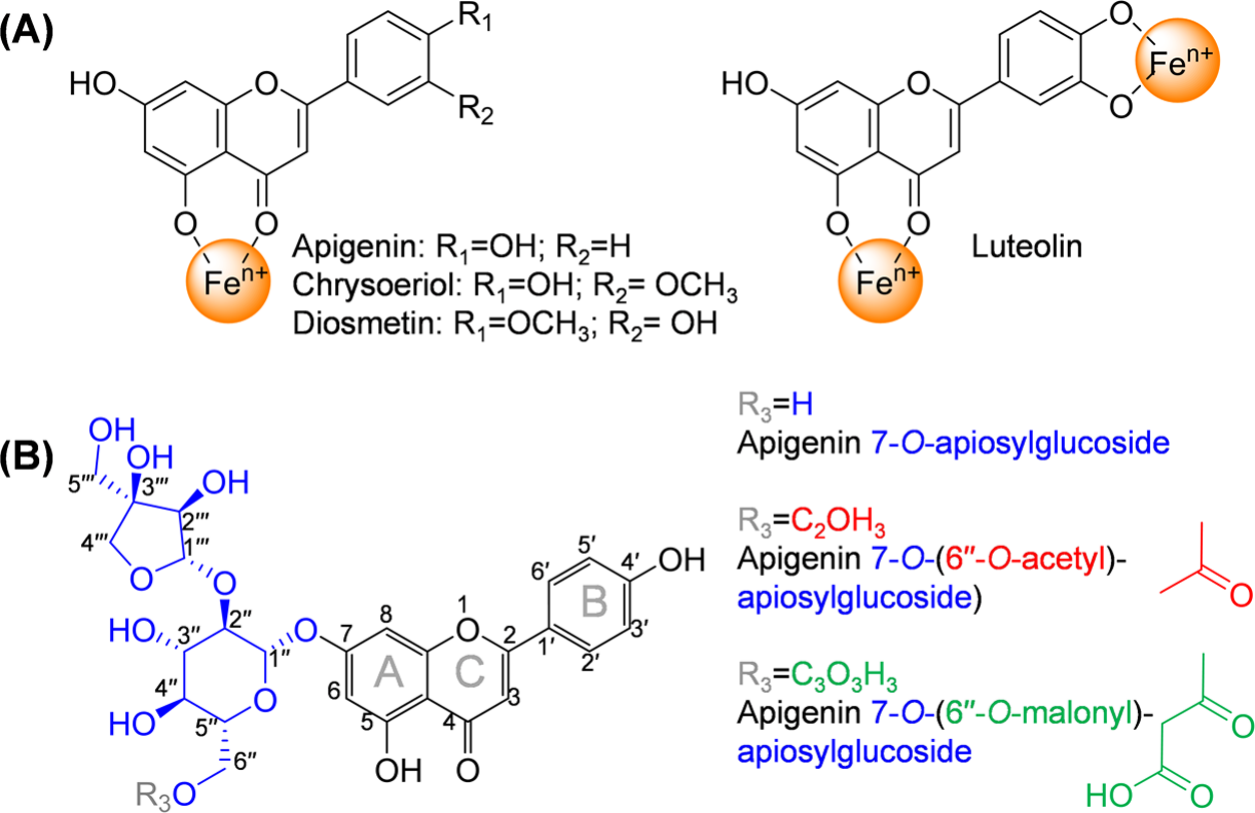
(A) Structures of common flavone aglycons present in celery and parsley including the proposed iron coordination to the 4–5 site for apigenin, chrysoeriol, and diosmetin and additional coordination to the 3′–4′ site for luteolin. (B) Structure of apigenin 7-*O*-apiosylglucoside (i.e., apiin), apigenin 7-*O*-(6″-*O*-acetyl)-apiosylglucoside (i.e., 6″-acetylapiin), and apigenin 7-*O*-(6″-*O*-malonyl)-apiosylglucoside (i.e., 6″-malonylapiin).^12,13^

Food fortified with iron can effectively reduce the global prevalence of iron deficiency.^1^ Bouillon and other types of savory concentrates are promising vehicles for iron fortification, as they are widely available, frequently consumed, and affordable.^2,3^ However, when bouillons are fortified with iron, the color and the bioavailability of iron can be compromised by the reactivity of the iron ion with phenolics.^4^ Bouillon typically contains salt, carbohydrates, starch, fats, proteins, herbs, and spices.^3^ The phenolics that react with iron mainly originate from the herbs and spices. Common herbs in bouillon cubes are parsley (*Petroselinium crispum*) and celery (*Apium graveolens*).^5^ These herbs are especially rich in flavones, a subclass of flavonoids that possess a 2-phenylchromen-4-one backbone (Figure 1A).^6^ Apigenin, chrysoeriol, diosmetin, and luteolin are examples of common flavone backbones that are present in celery and parsley.^7^ Iron can coordinate to the 5-hydroxy-4-ketone moiety (4–5 site) of flavones, as shown in Figure 1A. Additionally, iron coordination to the 3′–4′-dihydroxy moiety located in the B-ring (3′–4′ site) of flavones can occur, such as the B-ring of luteolin.^8^ In plants and plant-derived ingredients, the majority of flavones are glycosylated. Glycosylation of flavones reduces their reactivity and enhances their solubility in water.^9,10^ Glycosylated flavones from celery and parsley can also possess additional acylation of the glycoside moiety’s hydroxyl groups by acetyl or malonyl groups.^7,11−13^ So far, only the structures of apigenin 7-*O*-apiosylglucoside (also known as apiin), apigenin 7-*O*-(6″-*O*-acetyl)-apiosylglucoside (also known as 6″-acetylapiin), and apigenin 7-*O*-(6″-*O*-malonyl)-apiosylglucoside (also known as 6″-malonylapiin) have been confirmed by nuclear magnetic resonance (NMR) (Figure 1B).^12,13^ For the other (acylated) flavone glycosides in celery and parsley, identification was tentative and based solely on mass spectrometry.^7,11^ Detailed structural elucidation of these other (acylated) flavone glycosides has to be performed to confirm these tentative identifications.

To date, all research on understanding iron–phenolic interactions (i.e., complexation, oxidation, formation of networks) and its resulting discoloration in fortified foods has focused on model systems using simple phenolic and/or flavonoid aglycons.^8,14−18^ These studies show that flavonoid aglycons can form complexes with ferrous [Fe(II)] or ferric [Fe(III)] iron. Due to the higher stability of the Fe(III) complexes, the complexes with Fe(II) autoxidize to form colored Fe(III) iron complexes.^15^ Moreover, the complexation of Fe(III) to flavonoids can lead to oxidative coupling or oxidative degradation of flavonoids and the formation of iron–phenolic networks.^8^

The influence of 7-*O*-glycosylation and additional acylation on the interactions of flavones with iron is not yet understood. To be able to extrapolate the knowledge obtained using studies on flavonoid aglycons to real food fortification vehicles, such as bouillon, it is important to investigate the effect of flavone glycosylation and additional acylation on iron interaction. It has already been shown that 3-*O* and 5-*O* glucosylation affects metal coordination to the anthocyanin subclass of flavonoids and quercetin.^19−24^ However, because the 7–OH group of the flavone is not normally involved in iron complexation, it is hypothesized that 7-*O*-glycosylation of the flavone does not affect iron complexation and oxidation reactions. On the other hand, the addition of a malonyl group to the glycoside could potentially increase the stability of metal-flavonoid complexes due to the coordination of iron by the free carboxylate group.^21^ For acetylation, no such effect on complexation with iron is expected since there is no free carboxylate group.

This study aims to comprehensively investigate the effect of 7-*O* substitution of different flavone backbones with (acylated) apiosylglucosyl moieties. The flavones that are naturally present in bouillons and herbs were isolated and purified by methanolic extraction and preparative chromatography, respectively. Structural elucidation was performed by employing ion trap mass spectrometry (ITMS), high-resolution Orbitrap MS (FTMS), and nuclear magnetic resonance (NMR) spectroscopy. Subsequently, these purified compounds were incubated with iron(II) sulfate (FeSO_4_), and the influence of the flavones’ structural features on their interaction with iron in terms of complexation, oxidation, and discoloration was assessed via spectrophotometric and mass spectrometric techniques.

## 2 Materials and Methods

### 2.1 Materials

From a local supermarket chicken bouillon cubes (Knorr, Unilever) were purchased, containing the following ingredients as declared on the label: salt, vegetable fat (palm, shea butter, salt butter), flavor enhancer (E621, E627, E631), potato starch, sugar, onion powder, chicken fat (2%) (chicken fat, antioxidant E392), spices (turmeric, celery seed), carrot 1%, yeast extract, parsley, aroma, chicken meat extract (0.1%), caramel syrup, maltodextrin. Dried celery leaves (*A. graveolens*, Apiaceae) and dried parsley leaves (*P. crispum*, Apiaceae) (Verstegen Spices and Sauces BV) were also purchased at a local supermarket. Iron(II) sulfate heptahydrate (≥99 wt %), 3-(2-pyridyl)-5,6-diphenyl-1,2,4-triazine-*p*,*p*′-disulfonic acid monosodium salt hydrate (≥97 wt %; ferrozine), formic acid (≥98 vol %), and deuterium oxide (D_2_O) were obtained from Merck Life Science (Darmstadt, Germany). Luteolin (≥98 wt %) was purchased from Santa Cruz Biotechnology, Inc. (Santa Cruz, California), apigenin (≥98 wt %) from Indofine Chemical Company (Hillsborough, New Jersey), and chrysoeriol (≥95 wt %) from Extrasynthese (Genay, France). Ascorbic acid (≥99 wt %) was obtained from VWR International (Radnor, Pennsylvania). Dimethylsulfoxide (DMSO) was obtained from Merck Millipore (Billerica, Massachusetts). DMSO-*d*_6_ was purchased from Euriso-top (Saint-Aubin, France). ULC-MS grade acetonitrile (ACN) and water, both containing 0.1 vol % formic acid, and *n*-hexane (≥99 vol %) were purchased from Biosolve (Valkenswaard, The Netherlands). Water for other purposes than UHPLC was prepared using a Milli-Q water purification system (Merck Millipore, Billerica, Massachusetts).

### 2.2 Extraction of Phenolics from Bouillon and Herbs

Phenolics were extracted from the chicken bouillon and dried herbs. First, 2.5 g of bouillon or dried herb was ground with a mortar and pestle and defatted by extraction with 50 mL of *n*-hexane. To assist the extraction, the samples were vortexed and incubated in an ultrasonic bath (15 min, RT). Subsequently, the samples were centrifuged (10 min, 5000*g*), the *n*-hexane supernatants were discarded, and the pellets were subjected to two more identical extraction cycles. Any remaining *n*-hexane was removed from the pellet by flushing it with nitrogen. The defatted pellets were subjected to four extraction cycles, each with 50 mL of methanol (MeOH), using the same procedure as described for *n*-hexane. MeOH was evaporated by flushing with nitrogen, and the MeOH extracts were diluted to 1 mg mL^–1^ prior to analysis by reversed-phase ultrahigh-performance liquid chromatography coupled to electrospray ionization ion trap mass spectrometry (RP-UHPLC-PDA-ESI-ITMS*^n^*; Section 2.6).

### 2.3 Large-Scale Extraction of Phenolics from Celery

Lyophilized celery leaves were bead-milled (Cryomill MM440; Retsch GmbH, Haan, Germany) into a fine powder with stainless steel beads (ø 20 mm) at a frequency of 30 s^–1^, for 30 s. For the large-scale extraction, the extraction conditions were optimized and the celery powder was extracted with 80 vol % MeOH (5 mL g^–1^), as described elsewhere.^25^ Samples were subjected to sonication for 15 min. The suspensions were filtered over a paper filter under reduced pressure and the retentate celery powders were subjected to four more identical extraction cycles. The MeOH extracts were combined and concentrated to 25% of the initial volume under reduced pressure. Liquid–liquid partitioning with *n*-hexane (*n*-hexane/concentrated extract, 1:2.5 [*v*:*v*]) was performed three times to remove lipids and chlorophyll. The remaining MeOH in the cleaned MeOH extracts was evaporated under reduced pressure. Samples were resolubilized using *tert*-butanol and lyophilized to yield the cleaned extracts. Cleaned extracts were bead-milled (ø 20 mm beads, 30 s^–1^, 30 s).

### 2.4 Pre-Purification and Purification by Preparative RP-HPLC

Cleaned extracts were pre-purified with a Büchi Pure C-850 FlashPrep system, operated in flash mode, and equipped with a UV detector (Büchi, Flawil, Switzerland). Fractionation was performed on a Büchi FlashPure C18 cartridge (column size 80 g; particle size 40 μm) that was eluted with water (A) and acetonitrile (B), both acidified with 1 vol % formic acid, at room temperature at a flow rate of 60 mL min^–1^. The settings and elution profiles can be found in the Supporting Information (Method SI-1). The pre-purified pools were subjected to evaporation under reduced pressure to remove acetonitrile and were subsequently resolubilized using *tert*-butanol prior to lyophilization. Four flavone-enriched pools were further separated by preparative RP-HPLC, as described in the Supporting Information (Method SI-2), to obtain nine purified compounds. The purified compounds were analyzed by reversed-phase ultrahigh-performance liquid chromatography coupled to either electrospray ionization ion trap mass spectrometry or electrospray ionization hybrid quadrupole Orbitrap mass spectrometry (RP-UHPLC-PDA-ESI-ITMS*^n^* or RP-UHPLC-PDA-ESI-FTMS^2^), and nuclear magnetic resonance (NMR) spectroscopy (Section 2.6).

### 2.5 Incubation of Flavones with Iron

Stock solutions of each flavone were prepared by dissolving them in DMSO to a 20 mM concentration. Subsequently, each flavone stock solution was diluted either in water (flavone blank; final concentration flavone 1 mM; pH 6.0–7.5) or a freshly prepared FeSO_4_ solution (iron–flavone; final concentration flavone 1 mM; final concentration Fe(II) 1 mM; pH 4.5–5.0). The final volume of all samples was 1.50 mL, containing 5 vol % DMSO. Samples were placed in a 15 mL Greiner tube, and no measures were taken to reduce oxygen levels in the headspace. Aliquots of 0.25 mL were taken from the samples immediately after adding the flavone (*t*_0′_). Subsequently, each sample was adjusted to pH 6.5, and during the experiment, the pH was maintained at 6.5 by titration with 0.05 M HCl and 0.05 M NaOH using a pH-stat device (Metrohm, Herisau, Switzerland). This approach of maintaining pH with concentrated HCl and NaOH was previously used,^8^ and is preferred over the use of buffers to minimize interference of buffer compounds with complexation and oxidation reactions. After pH adjustment to 6.5 (*t*_0_), the samples were incubated at 40 °C under magnetic stirring (300 rpm) and aliquots (0.25 mL) were taken after 24 h (*t*_24_). Immediately after sampling, the samples were frozen in liquid nitrogen to stop any reactions. Samples were thawed on the day of analysis. The 0.25 mL samples were centrifuged (5 min, 15,000*g*) and the supernatants were separated to obtain the water-soluble (WS) fraction. The pellets were solubilized in DMSO and centrifuged once more (5 min, 15,000*g*) and the supernatants were separated to obtain the DMSO-soluble (DS) fraction. The DMSO insoluble pellets were freeze-dried to remove the remaining DMSO. Subsequently, 100 μL of 25 mM aqueous ascorbic acid was added to the freeze-dried pellets, which were then sonicated for 15 min, diluted 20 times with DMSO for a final DMSO concentration of 95 vol %, and sonicated for an additional 15 min. After sonication, the samples were centrifuged (5 min, 15,000*g*) and the supernatants were collected as the ascorbic acid-soluble (AAS) fractions. The remaining pellets were not analyzed further. DS, WS, and AAS fractions were immediately analyzed by RP-UHPLC-PDA-ESI-ITMS (Section 2.6). Quantification of the recovery of each flavone in the WS, DS, and AAS fraction was performed based on PDA peak area (280 nm) and a calibration curve of the corresponding purified flavone (0.03–1 mM, in duplicate, *R*^2^ ≥ 0.99). The relative quantity of flavone over time was defined as recovery, in which the starting concentration of flavone (1 mM) was set as 100%. To test if the trend in flavone decrease over time was statistically significant, analysis of variance (ANOVA) was performed using IBM SPSS Statistic v23 software (SPSS, Inc., Chicago, Illinois). Tukey’s post hoc comparisons (significant at *p* < 0.05) were carried out to evaluate differences per time point in the total flavone recovery and recovery of flavone in the WS, DS, and AAS fraction.

### 2.6 Identification of Phenolics

#### 2.6.1 RP-UHPLC-ESI-ITMS/FTMS Analysis

The extracts, pools, purified flavones, and flavones after incubation with iron were analyzed using a Thermo Vanquish UHPLC system (Thermo Scientific, San Jose, California). The UHPLC was equipped with an autosampler, a pump, a degasser, and a photodiode array (PDA) detector. The UHPLC was coupled to an LTQ Velos Pro ion trap mass spectrometer (ESI-ITMS*^n^*) or a Thermo Q Exactive Focus hybrid quadrupole Orbitrap mass spectrometer (ESI-FTMS^2^). The injection volume, column temperature, gradient elution program, and MS settings were used as described in the Supporting Information (Method SI-3).

#### 2.6.2 NMR Spectroscopy

The purified compounds were dissolved in DMSO-*d*_6_ (1 mg/0.5 mL). NMR spectra of the purified compounds were recorded on a Bruker Avance-III-700 spectrometer at a probe temperature of 300 K. ^1^H, HMBC, and HSQC spectra were acquired. Due to their limited solubility in DMSO-*d*_6_ and various other deuterated solvents, no HMBC and HSQC NMR spectra were obtained for compounds with a luteolin backbone. For luteolin 7-*O*-(6″-*O*-malonyl)-apiosylglucoside in D_2_O/DMSO-*d*_6_, the ^1^H spectrum could be acquired, which was solely used for purity determination.

### 2.7 Monitoring Complexation, Oxidation, and Discoloration by UV–Vis Spectroscopy

The effect of FeSO_4_ addition on complexation and oxidation reactions and their effect on discoloration was monitored using UV–vis spectroscopy. The UV–vis spectra of the WS and DS fractions were obtained after sampling and centrifugation, samples (50 μL) were diluted two times in water (WS fraction) or DMSO (DS fraction) and transferred to a Corning UV-transparent flat bottom polystyrene 96-well plate (Sigma-Aldrich, St. Louis, Missouri). Spectra were recorded in the range from 230 to 750 nm in a SpectraMax iD3 (Molecular Devices, Sunnyvale, California) at room temperature. The combined absorbance spectra (WS and DS) were normalized to the maximum absorption intensity. The color of the samples was recorded and assessed by spectrophotometric analysis and by taking pictures (OnePlus 7T, Shenzhen, China) using a ring light to cast a uniform light onto the subject against a white background.

### 2.8 Determination of Iron Concentration in Solution by Ferrozine-Based Colorimetric Assay

The total amount of iron in the WS, DS, and AAS fractions obtained at the different time points was quantified using a ferrozine-based colorimetric assay.^26^ Binding of ferrous iron by ferrozine results in the formation of a ferrous-ferrozine complex with λ_max_ at 565 nm.^27^ To ensure the reduction of ferric iron to its ferrous state, an excess of ascorbic acid (50 μL, 100 mM) was added to 50 μL of sample (i.e., WS, DS, AAS fractions). After 1 h incubation, an excess of ferrozine (50 μL, 40 mM) was added. Samples were transferred to 96-well microplates and the absorbance at 565 nm was measured in a SpectraMax iD3 at room temperature. All measurements were performed in duplicate. Quantification of total iron was performed based on a calibration curve of FeSO_4_ (0.00625–0.5 mM, in duplicate, *R*^2^ ≥ 0.99). Measurements were corrected for the flavone blank, and it was confirmed that the presence of DMSO did not interfere with the quantification of total iron.

## 3 Results and Discussion

### 3.1 Purification and Structural Elucidation of (Acylated) Flavone Glycosides

Eleven (acylated) flavone glycosides, six (acylated) phenolic acid glycosides, and three curcuminoids were tentatively identified in chicken bouillon methanol extract by RP-UHPLC-PDA-MS (Supporting Information, Figure SI-1 and Table SI-1). The chromatographic profile of the extract of bouillon showed similarities with the extracts of celery and parsley, which are the main herbs present in the bouillon cubes. To get more insight into the effect of flavone glycosylation and acylation on the interaction of flavones with iron, these compounds had to be purified. Phenolic acids, especially those with a catechol moiety, and curcuminoids may also form dark-colored complexes with Fe(III).^14,28^ However, only one minor peak in the bouillon extract was identified as a phenolic acid that possesses a catechol moiety (i.e., caffeoyl quinic acid); thus, it was not expected that the phenolic acids affect discoloration. As the focus of this work was on the effect of flavone glycosylation and additional acylation, the phenolic acids and curcuminoids were not further purified. The flavones were purified from celery extract as its flavone profile was most similar to that of bouillon extract and it contained fewer impurities than the bouillon extract itself (Supporting Information, Figure SI-1). Pre-purification by flash chromatography yielded four pools enriched in flavones (Supporting Information, Figure SI-2). Further purification by preparative RP-HPLC yielded nine purified flavones.

The purity and structure of the purified compounds were further elucidated by ITMS and FTMS, of which the spectrometric and spectroscopic data are shown in Table 1. The peaks were tentatively annotated based on the UV–visible absorbance (λ_max_), the exact mass of the parent ions, product ions in positive and negative modes, and a comparison of these data with the literature.^7,10,29^ Based on the product ions in CID MS^2^, the purified compounds consisted of three different flavone backbones: apigenin, luteolin, and chrysoeriol or diosmetin. Neutral losses (NL) of 132 amu (i.e., pentose) and 294 amu (i.e., pentose + hexose) in the MS^2^ fragmentation spectra indicate substitution with a pentosylhexoside (Supporting Information, Figure SI-3).^29^ Common glycosylation positions of flavones are *C*6, *C*8, or *O*7. Formation of ions with NL of 294 amu upon fragmentation indicates preferential cleavage at the glycosidic bond rather than cross-ring cleavage of the glycosyl moiety, thereby confirming *O*-glycosylation.^10^ In a previous study on parsley, the glycosides of apigenin were confirmed to be apiosylglucosides.^12^ Because parsley and celery both belong to the Apiaceae family and based on the neutral losses observed in UHPLC-MS, it is suggested that the pentosylhexoside substitutions on celery flavones are also apiosylglucosides.^7,30^ The additional neutral losses of 42 and 44 amu that were observed in the MS^2^ spectra of the acylated flavone glycosides (Supporting Information, Figure SI-3) are indicative for the substitution with acetyl or malonyl.^10^ The selective loss of apiose as evidenced by an NL of 132 in the MS^2^ data of the acetylated flavones confirms that acylation is not occurring on the apiosyl moiety and must, therefore, occur on the glucosyl moiety (Supporting Information, Figure SI-3).

**Table 1:** Purity, Spectrometric, and Spectroscopic Data of the Purified Compounds as Determined by UHPLC-PDA Coupled to ESI-ITMS or ESI-FTMS, and ^1^H NMR

									purity (%)^b^		
compound name	λ_max_ (nm)	molecular formula	ion	*m*/*z* calcd	*m*/*z* obs	error (ppm)	CID MS^2^ product ions (r.a.)^a^	HCD MS^2^ product ions (r.a.)	UV_280_	MS (NI)	^1^H NMR
apigenin 7-*O*-apiosylglucoside (apiin)	266, 338	C_26_H_28_O_14_	[M – H]^−^	563.14063	563.14044	–0.34	269, 431 (19)	269.04535	100	96	92
			[M + H]^+^	565.15519	565.15564	0.80	271, 433 (67)	271.06073			
apigenin 7-*O*-(6″-*O*-acetyl)-apiosylglucoside (6″-acetylapiin)	266, 338	C_28_H_30_O_15_	[M – H]^−^	605.15120	605.15106	–0.23	269, 563 (38), 473 (28)	269.04532	99	99	90
			[M + H]^+^	607.16575	607.16559	–0.27	271, 475 (96)	271.06046			
apigenin 7-*O*-(6″-*O*-malonyl)-apiosylglucoside (6″-malonylapiin)	266, 338	C_29_H_30_O_17_	[M – H]^−^	649.14103	649.14154	0.79	605	269.04535	88	86	81
			[M + H]^+^	651.15558	651.15497	–0.94	271, 519 (81)	271.06088			
chrysoeriol 7-*O*-apiosylglucoside	250, 350	C_27_H_30_O_15_	[M – H]^−^	593.15120	593.15106	–0.23	299, 284 (23), 461 (14)	299.05582, 284.03232 (15)	94	87	82
			[M + H]^+^	595.16575	595.16486	–1.50	301, 463 (46)	301.07126			
chrysoeriol 7-*O*-(6″-*O*-acetyl)-apiosylglucoside	250, 346	C_29_H_32_O_16_	[M – H]^−^	635.16176	635.16144	–0.51	299, 593 (59), 284 (31), 575 (15), 503 (15)	299.05585, 284.03232 (20)	99	98	90
			[M + H]^+^	637.17632	637.17639	0.12	301, 505 (77)	301.07092			
chrysoeriol 7-*O*-(6″-*O*-malonyl)-apiosylglucoside	250, 346	C_30_H_32_O_18_	[M – H]^−^	679.15159	679.15192	0.48	635	299.05582, 284.03226 (12)	87	79	76
			[M + H]^+^	681.16615	681.16724	1.61	301, 549 (60)	301.07135			
luteolin 7-*O*-apiosylglucoside	254, 350	C_26_H_28_O_15_	[M – H]^−^	579.13555	579.13562	0.12	285, 447 (75)	285.04013, 579.13525 (29), 447.09311 (10)	96	95	n.d.
			[M + H]^+^	581.15010	581.15009	–0.02	287, 449 (63)	287.05576			
luteolin 7-*O*-(6″-*O*-acetyl)-apiosylglucoside	250, 346	C_28_H_30_O_16_	[M – H]^−^	621.14611	621.14655	0.70	489, 285 (65), 579 (23)	285.04019, 621.14600 (23) 489.10364 (10)	45	50^c^	n.d.
			[M + H]^+^	623.16067	623.16144	1.24	287, 491 (89)	n.d.			
luteolin 7-*O*-(6″-*O*-malonyl)-apiosylglucoside	250, 346	C_29_H_30_O_18_	[M – H]^−^	665.13594	665.13641	0.70	621	285.04007, 621.14569 (28), 489.10342 (11)	90	66^d^	65
			[M + H]^+^	667.15050	667.14899	–2.26	287, 535 (80)	287.05518			

n.d., not defined; CID, collision-induced dissociation; HCD, higher-energy collisional dissociation.

a r.a., relative abundance. The threshold for fragments was ≥10%. The most intense fragment is underlined.

b UV_280_, purity expressed as a percentage of total peak area in UHPLC-PDA at 280 nm; MS (NI), purity expressed as a percentage of total peak area in UHPLC-ESI-MS negative ionization mode; ^1^H NMR, purity expressed as a percentage of the total peak area of the aromatic region in proton NMR spectroscopy.

c Main impurities are apigenin glucoside and apigenin malonyl glucoside.

d Main impurity is luteolin 7-*O*-apiosylglucoside.

Moreover, the initial annotations based on UHPLC-MS were verified by two-dimensional (2D) NMR using HMBC and HSQC (Supporting Information, Figures SI-4–SI-10). NMR spectra provided additional proof of glycosylation on the *O*7 position since the signals of *H*6 and *H*8 were present with a downfield shift compared to the flavone aglycon (Supporting Information, Figure SI-4). Additionally, the downfield shift of the *H*6″ upon acylation also indicated that acetyl and malonyl were linked to *O*6″ of the glucosyl moiety. These spectral features are in line with previously reported results for apigenin 7-*O*-(6″-*O*-acetyl)-apiosylglucoside and apigenin 7-*O*-(6″-*O*-malonyl)-apiosylglucoside.^12^ For the methoxylated flavone, the chemical shifts of *C*5′ (Supporting Information, Figures SI-8–SI-10) confirmed that the methoxy group was present on the *C*3′ position (i.e., chrysoeriol) and not on the *C*4′ position (i.e., diosmetin).^31^

Annotations of the compounds with a luteolin backbone could not be confirmed by NMR due to their limited solubility. Considering the identification of the other apigenin- and chrysoeriol-glycosides, and the fact that the putative luteolin glycosides are produced via the same biosynthetic pathways, their structural elucidation based on ITMS and FTMS is very likely. The purity of all purified compounds was determined by UV_280_, MS in negative ionization mode, and the aromatic region in the proton NMR spectrum and is shown in Table 1. Most purified compounds were very pure (∼90%) except for luteolin 7-*O*-(6″-*O*-acetyl)-apiosylglucoside and luteolin 7-*O*-(6″-*O*-malonyl)-apiosylglucoside. Nevertheless, these compounds still have respective purities of ∼65% and ∼50%, with the impurities being other (acylated) flavone glycosides. Thus, these compounds could be used to obtain a better insight into the interaction with iron, as long as the impurity is taken into account for data interpretation. To conclude this section, purification yielded nine differentially (acylated) flavone glycosides that were used in the next part of this study to identify the effect of flavone 7-*O*-glycosylation and 6″-*O*-acylation on the interactions with iron.

### 3.2 Effect of Substitution and Flavone Backbone on Recovery and Solubility

**Figure 2:**
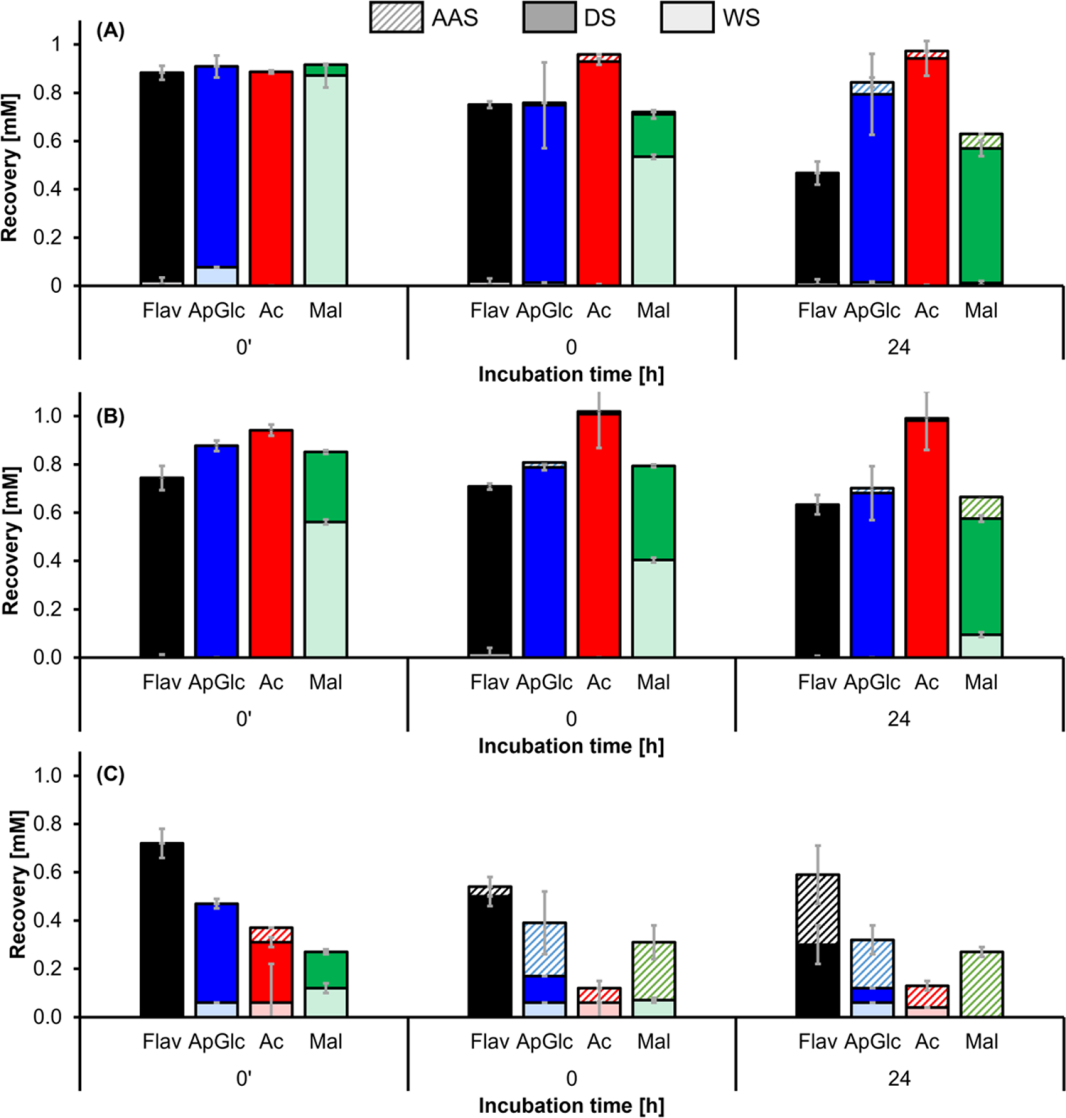
Recovery of the flavone aglycons (Flav; black) and apiosylglucoside (ApGlc; blue) with additional acetylation (Ac; red) or malonylation (Mal; green) for (A) apigenin, (B) chrysoeriol, and (C) luteolin in the water-soluble (WS), DMSO-soluble (DS), and ascorbic acid-soluble (AAS) fractions in the presence of equimolar concentration FeSO_4_. Time points shown are before the adjustment of the pH (*t*_0′_) and after 0 or 24 h of incubation at pH 6.5 in an aqueous solution. Error bars indicate the standard deviation of independent duplicates. The significance (Tukey’s test, *p* < 0.05) of differences in the total recovery is indicated in the Supporting Information, Table SI-2.

The purified (acylated) flavone glycosides and their commercial aglycons were incubated in the aqueous solution in the absence and presence of FeSO_4_ (equimolar concentration) at pH 6.5. The recovery of flavone in the water-soluble (WS), DMSO-soluble (DS), and ascorbic acid-soluble (AAS) fraction in the presence of equimolar concentration FeSO_4_ before pH adjustment (*t*_0′_), after adjustment of the pH to 6.5 (*t*_0_), and after incubation for 24 h at 40 °C (*t*_24_) was quantified by RP-UHPLC-PDA-MS (Figure 2). For the blank flavones in the absence of iron, it was observed that the order of water solubility was malonyl apiosylglucoside > acetyl apiosylglucoside > apiosylglucoside > aglycon (Supporting Information, Figure SI-11A). Interestingly, after 24 h incubation of the flavones in the absence of iron, most of the (acylated) flavone glycosides were recovered in the pellet (DS) and no longer in the supernatant (WS) (Figure SI-11A). The precipitation of these acylated glycosylated flavones in water over this time indicates the possible formation of larger self-associated aggregates or micelle-like aggregates because of their pronounced hydrophobic and hydrophilic moieties (Supporting Information, Figure SI-11B). The recovery of malonylated glycosylated flavones in the WS fraction was higher than that of the (acetylated) flavone glycosides. The negatively charged malonyl group is suggested to prevent the formation of self-associated or micellar-like aggregates via repulsion.

The addition of FeSO_4_ resulted in fast precipitation of the acylated flavone glycosides in water and a steep decrease in recovery for all flavones with the luteolin backbone (Figure 2). This decreased recovery after iron addition may be due to degradation reactions or the formation of insoluble metal–phenolic networks (MPNs), which is more likely for luteolin derivatives because the aglycon possesses two iron-binding sites, whereas apigenin and chrysoeriol only possess one iron-binding site.^8^ For the luteolin aglycon and its glycosides in particular, a significant proportion could be recovered in the AAS fraction, indicating that they are likely involved in the formation of MPNs. For the apigenin and chrysoeriol samples at 24 h, the malonylated flavone glycosides were also recovered in the AAS fraction. Malonylation is suggested to provide an extra iron-binding site and therefore allows for the formation of larger insoluble networks. Thus, the flavones are only recovered after the disruption of these networks by the addition of ascorbic acid.

Besides the solubility of the flavones, the total iron solubility [i.e., sum of Fe(II), Fe(III), and soluble products of Fe(II) and Fe(III)]^8^ was also assessed (Supporting Information, Figure SI-12). Before pH adjustment (*t*_0′_) and complex formation, iron was mainly recovered in the WS fractions. After pH adjustment (*t*_0_), the (acylated) flavone glycosides showed higher iron recovery in the WS fraction than the aglycons, for which most iron was recovered in the DS fraction. In line with the decreased water solubility of the flavones in the presence of iron over time, after 24 h, most of the iron was recovered in the DS or AAS fractions for all samples.

### 3.3 Color and Spectral Properties of (Acylated) Flavone Glycosides as Iron Complexes

**Figure 3:**
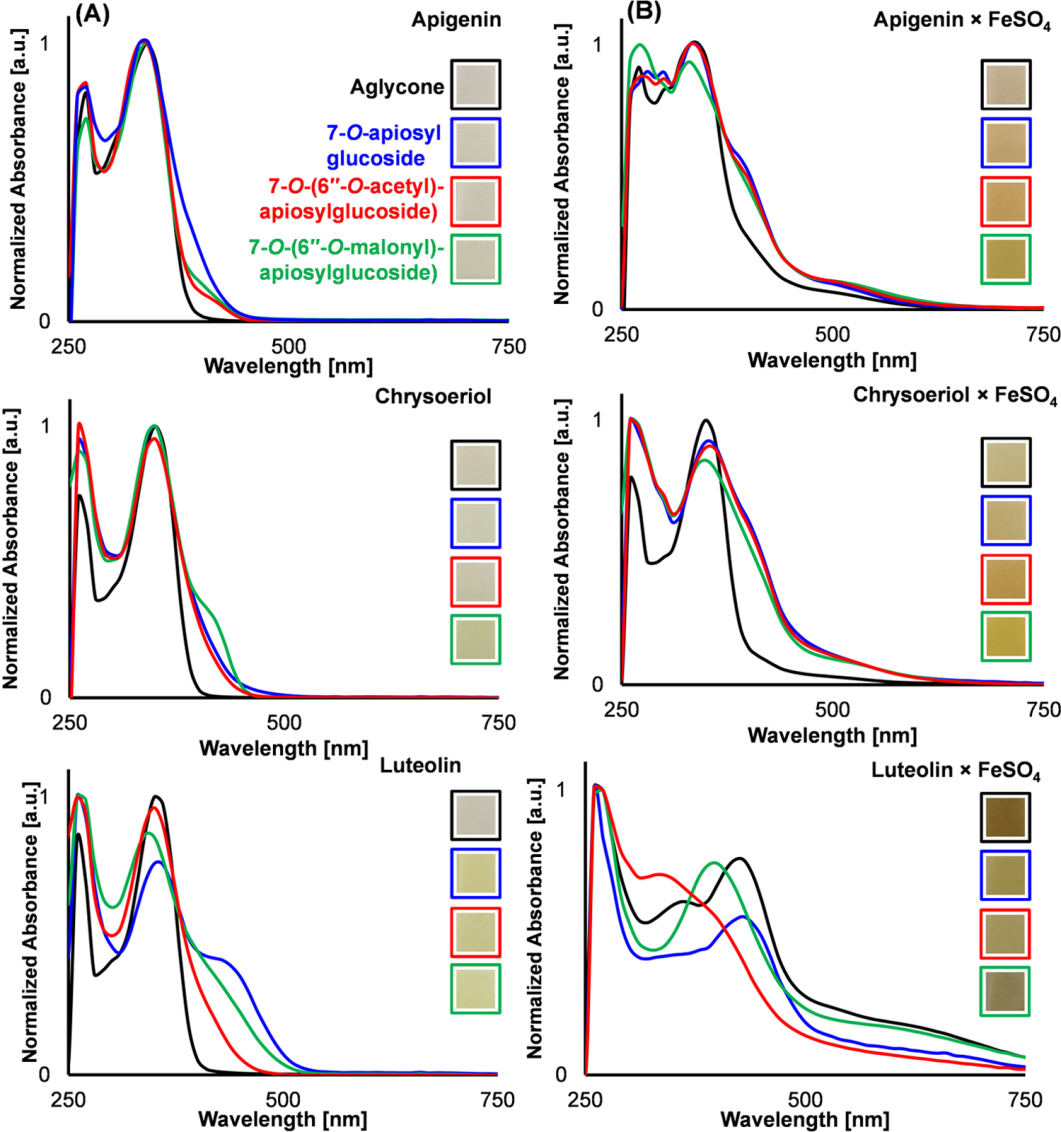
Normalized UV–vis absorbance spectra of the combined WS and DS spectra of the flavones at pH 6.5 (*t*_0_) in the (A) absence and (B) presence of FeSO_4_ at equimolar concentration. Insets show pictures of the aqueous solutions/dispersions of the flavones in the absence and presence of FeSO_4_ at pH 6.5. Separate absorbance spectra for the WS and DS fraction are shown in Figures SI-14 and SI-15 in the Supporting Information.

Absorbance spectra of the flavones in the absence or presence of FeSO_4_ (equimolar concentration) were obtained by UV–vis spectroscopy (Figure 3A). For the samples incubated in the absence of iron, it was observed that the λ_max_ values of the benzoyl (A-ring, 260–280 nm) and cinnamoyl (B-ring, 340–350 nm) bands were not affected by glycosylation and additional acylation. However, substitution did result in the formation of a shoulder around 400 nm. First, it was investigated whether this bathochromic shift was a result of self-association of the (acylated) flavone glycosides via π–π or CH−π stacking.^20,32^ However, the results in the Supporting Information (Figure SI-13) indicate that self-association was unlikely to be the underlying mechanism. It is more likely that this bathochromic shift is observed because the pH of the aqueous solution is close to the first p*K*_a_ of the flavone backbone,^33−35^ resulting in (partial) deprotonation of the (acylated) flavone glycosides in the WS fraction. Formation of a new band ∼ 400 nm was previously also observed for deprotonated species of other flavonoids.^36,37^ For the aglycons, this bathochromic shift was not observed because they were poorly soluble in water; thus, the spectra were measured in DMSO. The p*K*_a_ of the flavone backbone in DMSO is increased, due to the lower dielectric constant of DMSO compared to water.^38^ Therefore, the aglycons are fully protonated and no shoulder at 400 nm is observed. This reasoning was confirmed by measuring the flavones in 50 vol % aq ACN with 0.1 vol % formic acid, which resulted in identical UV–vis spectra of the aglycons and (acylated) flavone glycosides (data not shown).

The addition of equimolar concentration FeSO_4_ to the flavones and adjustment of the pH to 6.5 resulted in a bathochromic shift for all samples (Figure 3B). This bathochromic shift indicates that Fe(III)-flavone complexes are present in the solutions. Complexes of Fe(II) with phenolics in the absence of oxygen were previously demonstrated to show no bathochromic shift of the spectra to the visible range and were colorless.^39−41^ In the presence of oxygen these Fe(II)–phenolic complexes show fast autooxidation to Fe(III)–phenolic complexes because of the higher stability of the Fe(III)–phenolic complexes.^42,43^

For the samples with an apigenin or chrysoeriol backbone, the cinnamoyl band was shifted to the visible range, resulting in a light yellow-brown color (Figure 3B). For luteolin samples, additional formation of a broad absorbance band at 550–750 nm was observed due to ligand-to-metal charge transfer (LMCT), resulting in a darker brownish or blackish color (Figure 3B). It is expected that only the flavones with a luteolin backbone show an LMCT band because it is the only tested flavone backbone with a catechol moiety on the B-ring.^8^

Slightly more discoloration and a bathochromic shift of the cinnamoyl bands were observed for the (acylated) glycosides of apigenin and chrysoeriol samples in the presence of FeSO_4_ compared to the aglycons. All (acylated) glycosides and aglycons in the presence of FeSO_4_ were mainly recovered in the DS fraction (Figures 2, SI-14, and SI-15). Thus, the observed bathochromic shift is not a result of solvent effects, as described above for the samples in the absence of FeSO_4_ but is apparently caused by the presence of the (acylated) apiosylglucosyl moiety. To verify that the lower relative polarity of DMSO (0.44) in comparison to water (1.00) did not affect the electronic transitions and absorbance spectra, the WS and DS spectra for chrysoeriol 7-*O*-(6″-*O*-malonyl)-apiosylglucoside in the presence of FeSO_4_ were compared. These spectra were almost identical, thereby confirming that the impact of the selected solvents (water and DMSO) on the obtained spectra was minimal (Figure SI-16).

Interestingly, no difference in absorbance was observed between the acylated glycosides and the nonacylated glycosides. Contrary to the initial hypothesis that malonylation would affect the stability of iron-flavonoid complexes, this indicates that the increase in discoloration is solely due to the presence of the 7-*O*-apiosylglucosyl moiety and is not enhanced by additional malonylation. The increased discoloration and more pronounced bathochromic shift for the apigenin and chrysoeriol 7-*O*-apiosylglucosides, compared to the aglycon, indicates that the ability of the 4–5 site to coordinate iron at pH 6.5 is increased by 7-*O-*glycosylation.

For luteolin, less discoloration was observed for the (acylated) glycosides compared to the algycon. It should be noted that the acylated glycosides are of lower purity (Table 1) and may therefore show less absorbance. However, luteolin apiosylglucoside (≥95% purity) also showed a decrease in discoloration in the presence of FeSO_4_ compared to the aglycon, which indicates that the decrease in discoloration is due to the addition of (acylated) 7-*O*-apiosylglucosyl moieties and not due to lower purity. Besides the 4–5 site, the luteolin backbone also possesses the 3′–4′ site, which is generally regarded as the strongest Fe(III) binding site.^44^ Increased ability of iron to coordinate to the 4–5 site of luteolin 7-*O*-glycosides allows it to compete with coordination at the 3′–4′ site, which decreases discoloration.^8^ It is expected that when equimolar concentrations of iron and luteolin apiosylglucoside are present, a mixture of the 4–5 and the 3′–4′ complexes will exist in solution. This effect is expected to diminish at a 1:2 ratio of flavone:iron, as sufficient iron would be present to bind at the 3′–4′ site and 4–5 site simultaneously. However, upon testing this experimentally, much more precipitation was observed in both the DS and WS fraction, which hindered further in-depth analysis. Increased precipitation suggested that increasing the relative amount of iron leads to the formation of insoluble metal–phenolic networks.

### 3.4 Increased Iron Coordination to the Flavone 4–5 Site by 7-*O*-Glycosylation

There are several possible explanations why coordination to the 4–5 site is preferred for the flavone 7-*O*-apiosylglucosides compared to the aglycons. The first hypothesis was that the apiosyl residue can stabilize the iron bound to the 4–5 site of the flavone by additional coordination of the glycosyl −OH groups to iron. However, in an additional experiment a bathochromic shift was also observed for apigenin 7-*O*-glucoside, which lacks the apiosyl moiety, compared to the apigenin aglycon (results not shown). This indicates that the involvement of the apiosyl moiety is not the (sole) mechanism stabilizing iron bound to the 4–5 site. Another possible explanation for the increased ability of iron to coordinate to the 4–5 site is a lower p*K*_a_ of the 5–OH group due to 7-*O*-glycosylation and thus increased deprotonation of the 5–OH at pH 6.5, thereby enhancing its ability to coordinate iron. The p*K*_a_ can either be lowered due to the absence of the free 7–OH group or because glycosylation introduces a bulky, electron-withdrawing group on the *O*7 position, thereby making the 5–OH slightly more acidic.^22,45^ The last possible explanation for the increased ability of iron to coordinate to the 4–5 site is related to the lower planarity of the glycosylated flavones, compared to the flavone aglycons, which can decrease the hydrophobic π–π stacking interactions of the aromatic nuclei.^46^ The π–π stacking interactions play an important role in metal–ligand complexes.^47^ A decrease in stacking for the flavone 7-*O*-glycosides may potentially increase the ability of the 4–5 site to coordinate iron. It can be concluded that the ability of the 4–5 site to coordinate iron is increased by 7-*O*-glycosylation, yet the underlying mechanism remains unclear and should be investigated in future experimental studies, and confirmed by additional in silico modeling.

### 3.5 Effect of Glycosylation and Additional Acylation on Flavone Stability in the Presence of Iron

It is known that, depending on the flavonoids’ aglycon structural features, complexation with Fe(III) can be followed up by electron transfer reactions that cause oxidation of the flavonoid.^8,48^ The formation of degradation and polymerization products was investigated by RP-UHPLC-PDA-MS. The chromatograms of the flavones in the presence of FeSO_4_ before pH adjustment (*t*_0′_), after adjustment of the pH to 6.5, and after incubation for 24 h (*t*_24_) are shown in Supporting Information (Figure SI-17). None of the characteristic degradation products of iron-mediated oxidative degradation of flavonoid aglycons, such as 4-hydroxybenzoic acid, 3,4-dihydroxybenzoic acid, and 2,4,6-trihydroxyphenylglyoxilic acid,^8,48^ were found in extracted ion chromatograms of the samples after 24 h incubation. Moreover, no formation of other oxidative degradation products, dimers, or larger oxidative coupling products was observed in any of the obtained chromatograms, including full MS (negative and positive mode) and PDA (190–680 nm). The stability of the apiosylglucosyl, acetylapiosylglucosyl, and malonylapiosylglucosyl moieties was also investigated in the presence of iron as shown in Supporting Information (Figure SI-18). No deglycosylation was observed for any of the flavones in the absence or presence of iron. Deacetylation was observed in minor amounts (<1%) in the absence and presence of iron. For the malonylated flavone glycosides, 10–20% cleavage of the malonyl group was observed after 24 h incubation in an aqueous solution at pH 6.5, regardless of the presence of iron. Thus, these results indicate that iron did not affect deglycosylation, deacetylation, and demalonylation reactions. Additionally, glycosylation and additional acylation did not affect the degradation of the flavone backbone.

### 3.6 Effect of the Structural Features of Flavones on Their Interaction with Iron

**Figure 4:**
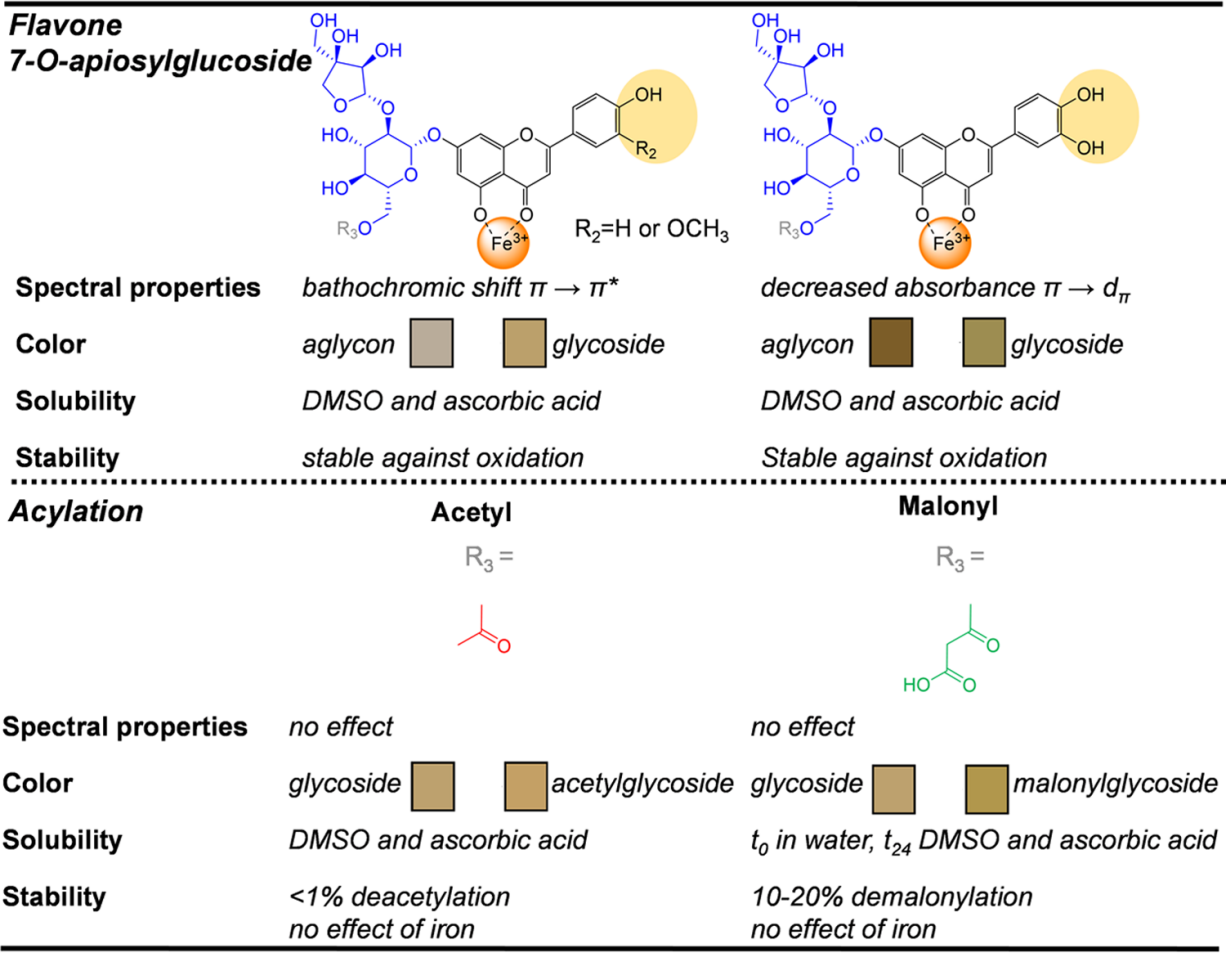
Structure–reactivity relationships highlighting the influence of flavone backbone, glycosylation, and additional acylation on the reactivity (i.e., complexation, oxidation, discoloration) with iron at equimolar concentration.

An overview of the effect of 7-*O*-apiosylglucosylation and additional 6″-*O*-acetylation or 6″-*O*-malonylation of the flavone backbone on its reactivity with iron is provided in Figure 4. Overall, the present findings show that 7-*O*-apiosylglucosylation of flavones affects the formed iron–flavone complex and the resulting color. For the flavones that only possess the 4–5 binding site (i.e., apigenin and chrysoeriol), more bathochromic shifting and discoloration was observed for the (acylated) glycosides compared to the aglycon due to an increased ability of iron to coordinate to the 4–5 site. At equimolar iron/flavone concentration, the increased ability of iron to coordinate to the 4–5 site of (acylated) luteolin glycosides competes with binding to the 3′–4′ site, reducing observed discoloration compared to the aglycon. This is the result of lower intensity of the LMCT absorbance band that is typically observed for iron–catecholate complexes.

In conclusion, the findings of this work indicate that 7-*O*-apiosylglucosylation of flavones affects their iron complexation behavior by increasing the ability of iron to coordinate to the 4–5 binding site. The presence of iron does not affect the oxidative degradation of the flavone backbone. These results demonstrate that outcomes of iron interaction studies with flavonoid aglycon model systems cannot be directly extrapolated to iron-fortified food systems containing (acylated) flavonoid glycosides. Thus, to understand discoloration in iron-fortified foods, model systems should also comprise (acylated) glycosides of relevant flavonoids. The presented results indicate that (acylated) flavone glycosides show less intense discoloration than what was previously reported for flavonoid aglycons and curcuminoids. Therefore, discoloration in fortified bouillons is most likely not only caused by the complexation of iron with natural flavones.

## Supporting Information Available

Pre-purification by RP-flash chromatography (Method SI-1); preparative RP-UHPLC-ESI-MS (Method SI-2); identification and quantification of phenolics in the extract by RP-UHPLC-PDA-ITMS/FTMS (Method SI-3); RP-UHPLC-UV profile (280 nm) of phenolic extracts (Figure SI-1); spectrometric and spectroscopic data of phenolic compounds in extracts (Table SI-1); RP-UHPLC-UV profile (280 nm) of celery extract and pools A–D (Figure SI-2); negative-mode CID MS^2^ fragmentation of purified compounds (Figure SI-3); 2D HSQC NMR spectra to confirm glycosylation and acylation position (Figure SI-4); 2D HSQC and HMBC spectrum of apigenin 7-*O*-apiosylglucoside (Fig. SI-5); 2D HSQC and HMBC spectrum of apigenin 7-*O*-(6″-*O*-acetyl)-apiosylglucoside (Figure SI-6); 2D HSQC and HMBC spectrum of apigenin 7-*O*-(6″-*O*-malonyl)-apiosylglucoside (Figure SI-7); 2D HSQC and HMBC spectrum of chrysoeriol 7-*O*-apiosylglucoside (Figure SI-8); 2D HSQC and HMBC spectrum of chrysoeriol 7-*O*-(6″-*O*-acetyl)-apiosylglucoside (Figure SI-9); 2D HSQC and HMBC spectrum of chrysoeriol 7-*O*-(6″-*O*-malonyl)-apiosylglucoside (Figure SI-10); recovery of flavones in the WS, DS, and AAS fractions (Figure SI-11); recovery of iron in the WS, DS, and AAS fractions (Figure SI-12); statistical analysis of flavone recovery (Table SI-2); absorbance at 400 nm of different concentrations acylated and glycosylated apigenins at pH 6.5 (Figure SI-13); UV–vis absorbance spectra of the WS fraction (Figure SI-14); UV–vis absorbance spectra of the DS fraction (Figure SI-15); UV–vis absorbance spectra of the WS and DS fraction of chrysoeriol 7-*O*-(6″-*O*-malonyl)-apiosylglucoside (Figure SI-16); RP-UHPLC-PDA chromatograms of (acylated) flavone glycosides (Figure SI-17); and stability of malonylated and acetylated flavone 7-*O*-apiosylglucosides (Figure SI-18) (PDF)

## Abbreviations

AAS: ascorbic acid-soluble
DS: DMSO-soluble
Fe(II): ferrous iron
Fe(III): ferric iron
FTMS: high-resolution Orbitrap mass spectrometry
HMBC: heteronuclear multiple bond correlation
HSQC: heteronuclear single quantum correlation
ITMS: ion trap mass spectrometry
MPN: metal–phenolic network
NL: neutral losses
NMR: nuclear magnetic resonance
PDA: photodiode array
RP-UHPLC: reversed-phase ultrahigh-performance liquid chromatography
UV–vis: ultraviolet–visible
WS: water-soluble
