# Supplementary material for: Interactions of Natural Flavones with Iron Are Affected by 7-O-Glycosylation, but Not by Additional 6″-O-Acylation
Source: ACS Food Sci Technol. 2023 May 2;3(6):1111–21. doi: 10.1021/acsfoodscitech.3c00112 (PMC10278068; doi:10.1021/acsfoodscitech.3c00112)
Supplement: Supplementary file 1 — fs3c00112_si_001.pdf [file fs3c00112_si_001.pdf]

Supporting Information

Interactions of natural flavones with iron are affected by 7-*O*-glycosylation, but not by additional 6″-*O*-acylation

Judith BIJLSMA[1], Wouter J.C. DE BRUIJN[1], Jamie KOPPELAAR[1], Mark G. SANDERS[1], Krassimir P. VELIKOV[2,3,4], and Jean-Paul VINCKEN[1,*]

[1] Laboratory of Food Chemistry, Wageningen University & Research, Bornse Weilanden 9, P.O. Box 17, 6700 AA, Wageningen, The Netherlands

[2] Unilever Innovation Centre Wageningen B.V. Bronland 14, 6708 WH Wageningen, The Netherlands

[3] Institute of Physics, University of Amsterdam, Science Park 904, 1098 XH Amsterdam, the Netherlands

[4] Soft Condensed Matter, Debye Institute for Nanomaterials Science, Utrecht University, Princetonplein 5, 3584 CC Utrecht, The Netherlands

* Corresponding author: jean-paul.vincken@wur.nl

Supporting Information with "Interactions of natural flavones with iron are affected by 7-*O*-glycosylation, but not by additional 6″-*O*-acylation" by Bijlsma, de Bruijn, Koppelaar, Sanders, Velikov, and Vincken.

**Contents**		**Page**
**Method S1-1**	Pre-purification by RP-flash chromatography	*S3*
**Method S1-2**	Preparative RP-UHPLC-ESI-MS	*S4*
**Method SI-3**	Identification and quantification of phenolics in the extract by RP-UHPLC-PDA-ITMS/FTMS	*S5*
**Fig. SI-1**	RP-UHPLC-UV profile (280 nm) of phenolic extracts	*S8*
**Table SI-1**	Spectrometric and spectroscopic data of phenolic compounds in extracts	*S9*
**Fig. SI-2**	RP-UHPLC-UV profile (280 nm) of celery extract and pools A-D	*S11*
**Fig. SI-3**	Negative mode CID MS^2 fragmentation of purified compounds	*S12*
**Fig. SI-4**	2D HSQC NMR spectra to confirm glycosylation and acylation position	*S15*
**Fig. SI-5**	2D HSQC and HMBC spectrum of apigenin 7-*O*-apiosylglucoside	*S16*
**Fig. SI-6**	2D HSQC and HMBC spectrum of apigenin 7-*O*-(6″-*O*-acetyl)-apiosylglucoside	*S17*
**Fig. SI-7**	2D HSQC and HMBC spectrum of apigenin 7-*O*-(6″-*O*-malonyl)-apiosylglucoside	*S18*
**Fig. SI-8**	2D HSQC and HMBC spectrum of chrysoeriol 7-*O*-apiosylglucoside	*S19*
**Fig. SI-9**	2D HSQC and HMBC spectrum of chrysoeriol 7-*O*-(6″-*O*-acetyl)-apiosylglucoside	*S20*
**Fig. SI-10**	2D HSQC and HMBC spectrum of chrysoeriol 7-*O*-(6″-*O*-malonyl)-apiosylglucoside	*S21*
**Fig. SI-11**	Recovery of flavones in the WS, DS, and AAS fractions	*S22*
**Fig. SI-12**	Recovery of iron in the WS, DS, and AAS fractions	*S23*
**Table SI-2**	Statistical analysis by Tukey's *post hoc* comparison of the flavone and iron recovery in total and in the fractions	*S24*
**Fig. SI-13**	Absorbance at 400 nm of different concentrations acylated and glycosylated apigenins at pH 6.5	*S26*
**Fig. SI-14**	UV-Vis absorbance spectra of the WS fraction	*S27*
**Fig. SI-15**	UV-Vis absorbance spectra of the DS fraction	*S28*
**Fig. SI-16**	UV-Vis absorbance spectra of the WS and DS fraction of chrysoeriol 7-*O*-(6″-*O*-malonyl)-apiosylglucoside	*S29*
**Fig. SI-17**	RP-UHPLC-PDA chromatograms of (acylated) flavone glycosides	*S30*
**Fig. SI-18**	Stability of the malonylated (A) and acetylated (B) flavone apiosylglucosides in presence or absence of iron ($FeSO_4$)	*S33*
**References**		*S34*

Method SI-1 Pre-purification by RP-flash chromatography

Pre-purification of the celery extract was performed by use of a Büchi Pure C-850 FlashPrep system, operated in flash mode and equipped with a UV detector (Büchi, Flawil, Switzerland). The cleaned celery extracts (4 g) were dissolved in 100% MeOH and 8 g of C18 bulk sorbent was added (Bondesil, particle size 40 μm). Subsequently, the MeOH was evaporated under reduced pressure. The remaining powder was dry-loaded in 15 g cartridges (Grace, Columbia, USA) on the FlashPrep system with a solid loader plunger. The fractionation was performed at room temperature on a Büchi FlashPure C18 cartridge (column size 80 g; particle size 40 µm). Water and ACN (ULC/MS grade), both acidified with 1 vol.% formic acid, were used as eluent A and B, respectively. The flow rate was 60 mL min^{-1} and UV detection was set from 254 to 400 nm. The following elution profile was used: 0 – 7.3 min, isocratic at 20 vol.% B; 7.3 – 36.6, linear gradient from 20 to 30 vol.% B; 36.6 – 38.1, linear gradient from 30 to 100 vol.% B; 38.1 – 45.4, isocratic at 100 vol.% B; 45.4 – 46.9, linear gradient from 100 to 20 vol.% B; 46.9 – 54.3, isocratic at 20 vol.% B. Several runs were performed to pre-purify a total of 44.5 g of celery extract. The collected fractions were analysed by RP-UHPLC-PDA-ESI-ITMS^n and those containing similar compounds were pooled. The ACN was removed under reduced pressure at 45 °C using a rotavapor (Büchi, Fawil, Switzerland) and the remaining water was removed by lyophilisation. Afterwards the flavone-enriched pools were solubilised in DMSO for analysis on RP-UHPLC-PDA-ITMS^n.

Method SI-2 Preparative RP-HPLC-ESI-MS

Purification by preparative HPLC

Purification of the flavone-enriched pools was performed using a Waters preparative HPLC, equipped with a 2545 quaternary gradient module, 2767 sample manager, fluid organizer, and 2998 photodiode array detector. The pools (~10 mg in 1 mL 20% ACN) were injected on a Waters XBridge Prep C18 OBD column (19 × 250 mm, 5 μm particle size) (Waters, Milford, MA, USA) and eluents used were water (A) and ACN (HPLC-R grade) (B), both acidified with 1 vol.% formic acid. The elution programs for the different pools were as follows:

Pool 1: Isocratic at 13 vol.% B for 5.65 min, linear gradient to 18 vol.% B from 5.65 to 40.48 min, linear gradient to 100 vol.% B from 40.48 to 43.97 min, isocratic at 100 vol.% B from 43.97 to 61.39 min, linear gradient to 13 vol.% B from 61.39 to 64.87 min, isocratic at 13 vol.% B from 64.87 to 82.29 min. **Pool 2**: Isocratic at 15 vol.% B for 5.65 min, linear gradient to 20 vol.% B from 5.65 to 40.48 min, linear gradient to 100 vol.% B from 40.48 to 43.97 min, isocratic at 100 vol.% B from 43.97 to 61.39 min, linear gradient to 15 vol.% B from 61.39 to 64.87 min, isocratic at 15 vol.% B from 64.87 to 82.29 min. **Pool 3**: Isocratic at 19 vol.% B for 5.65 min, linear gradient to 24 vol.% B from 5.65 to 40.48 min, linear gradient to 100 vol.% B from 40.48 to 43.97 min, isocratic at 100 vol.% B from 43.97 to 61.39 min, linear gradient to 19 vol.% B from 61.39 to 64.87 min, isocratic at 19 vol.% B from 64.87 to 82.29 min. **Pool 4**: Isocratic at 22 vol.% B for 5.65 min, linear gradient to 27 vol.% B from 5.65 to 40.48 min, linear gradient to 100 vol.% B from 40.48 to 43.97 min, isocratic at 100 vol.% B from 43.97 to 61.39 min, linear gradient to 22 vol.% B from 61.39 to 64.87 min, isocratic at 22 vol.% B from 64.87 to 82.29 min. The purified fractions were subjected to evaporation under reduced pressure to remove acetonitrile, and were subsequently resolubilized using *tert*-butanol prior to lyophilization.

Method SI-3 Identification and quantification of phenolics in the extract by RP-UHPLC-PDA-ITMS/FTMS

Samples were separated on a Thermo Vanquish UHPLC system (Thermo Scientific, San Jose, CA, USA) equipped with an autosampler, a pump, a photodiode array (PDA) detector. A sample (1 µL) was injected on an Acquity UPLC BEH C18 column (150 mm × 2.1 mm i.d., 1.7 µm) with a VanGuard (5 mm × 2.1 mm i.d., 1.7 µm) guard column of the same material (Waters, Milford, MA). Water (A) and acetonitrile (B), both acidified with 0.1 vol.% formic acid, were used as eluents. The flow rate was 400 µL min^{-1}, and the temperature of the column oven was 45 °C with the post column cooler set to 40 °C. Various elution profiles were used, depending on the type of sample. *For the extracts and the enriched pools:* 0.00 – 1.09 min, isocratic on 1 vol.% B; 1.09 – 33.26 min, linear gradient from 1 to 60 vol.% B; 33.26 – 34.35 min linear gradient from 60 to 100 vol.% B; 34.35 – 39.80 min isocratic on 100 vol.% B; 39.80 – 40.89 min linear gradient from 100 to 1 vol.% B; 40.89 – 46.35 min isocratic on 1 vol.% B. *For the purified compounds*: 0.00 – 1.09 min, isocratic on 1 vol.% B; 1.09 – 22.54 min, linear gradient from 1 to 55 vol.% B; 22.54 – 23.63 min linear gradient from 55 to 100 vol.% B; 23.63 – 29.08 min isocratic on 100 vol.% B; 29.08 – 30.17 min linear gradient from 100 to 1 vol.% B; 30.17– 35.62 min isocratic on 1 vol.% B. *For the reactivity of the purified compounds with iron*: 0.00 – 1.09 min, isocratic on 1 vol.% B; 1.09 – 20.72 min, linear gradient from 1 to 55 vol.% B; 20.72 – 21.81 min linear gradient from 55 to 100 vol.% B; 21.81 – 27.26 min isocratic on 100 vol.% B; 27.26 – 28.35 min linear gradient from 100 to 1 vol.% B; 28.35– 33.81 min isocratic on 1 vol.% B. The PDA detector was set to measure spectra in the wavelength range of 190 – 680 nm. The temperature of the autosampler was controlled at 10 °C, except the samples dissolved in DMSO, where the temperature was set at 25 °C to prevent solidification of DMSO (T_m = 18.5 °C).

Electrospray ionization ion trap mass spectrometry (ESI-ITMS^n)

Mass spectrometric data of the extracts, pools, and purified compounds were acquired using an LTQ Velos Pro linear ion trap mass spectrometer (Thermo Scientific) equipped with a heated electrospray ionization probe (ESI-ITMS^n) and coupled to the Vanquish UHPLC system. Nitrogen was used as a sheath gas (50 arbitrary units) and auxiliary gas (13 arbitrary units). Data were collected over the *m/z* range of 150 – 1,500 in negative and positive ionisation mode by using source voltages of 2.5 and 3.5 kV, respectively. For both modes, the S-lens RF level was set at 67%, the ion transfer tube temperature was 263 °C, and the source heater temperature 425 °C. Data-dependent MS^n analysis was performed on the most intense ion by collision-induced dissociation (CID) with normalised collision energy of 35%. A dynamic mass exclusion approach was used, in which the most intense ion was fragmented 3 times and was subsequently excluded from fragmentation for the following 5 seconds, allowing data-dependent MS^n of less intense co-eluting compounds. Data acquisition and processing were performed using Xcalibur version 4.1 (Thermo Scientific).

Electrospray ionization hybrid quadrupole Orbitrap mass spectrometry (ESI-FTMS^2)

Accurate mass data of the purified compounds were acquired using a Thermo Q Exactive Focus hybrid quadrupole-Orbitrap Fourier transform mass spectrometer (FTMS) (Thermo Scientific) equipped with a heated ESI probe coupled to the Vanquish UHPLC system. Prior to analysis, the mass spectrometer was calibrated in the negative and positive ionization mode using Tune 2.11 (Thermo Scientific) by injection of Pierce negative and positive ion calibration solutions (Thermo Scientific). Gas flows and source conditions were the same as for ESI-ITMS. Full MS and higher energy collisional dissociation (HCD) fragmentation data were recorded at 70.000 FWHM and 35.000 FWHM resolution, respectively. Normalized collision energy was 35%. MS^2 fragmentation was performed on the most intense product ion in the MS spectrum and the

exact masses of the purified compounds were inserted in an inclusion list. Data acquisition and processing were performed using Xcalibur version 4.1 (Thermo Scientific).

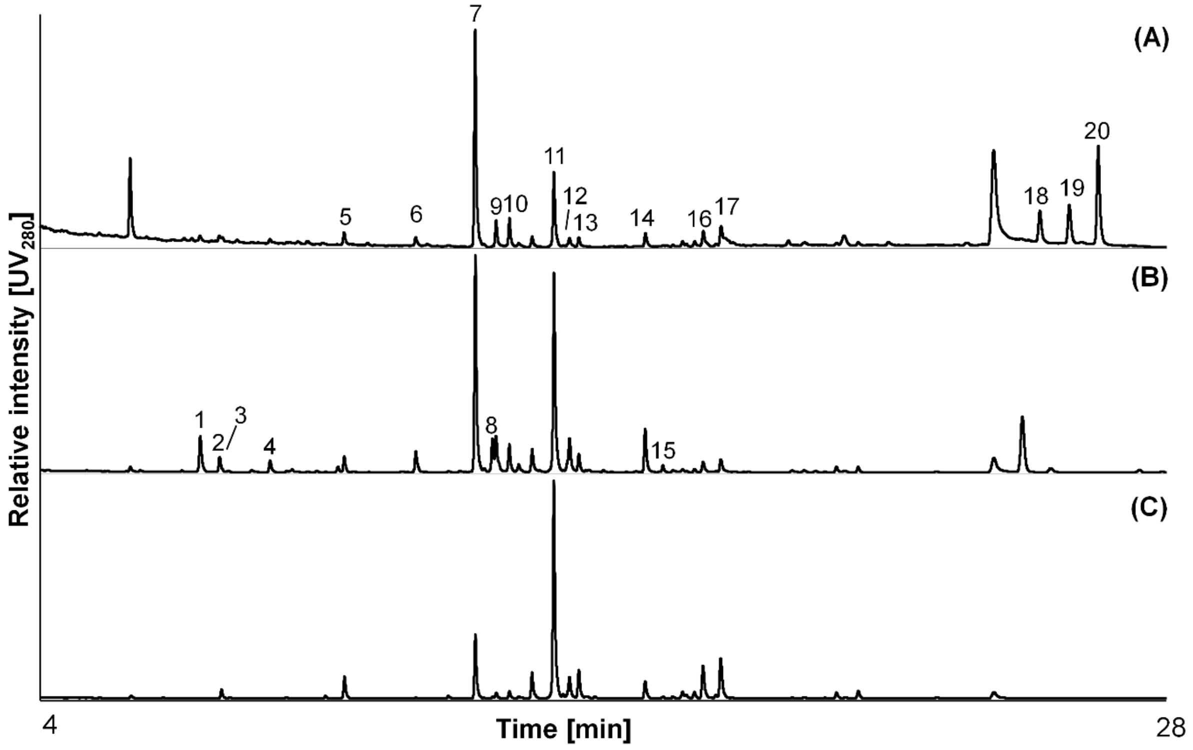

Figure SI-1. RP-UHPLC-UV profile (280 nm) of the phenolic extracts from chicken bouillon (**A**), celery (**B**), and parsley (**C**). Peak numbers refer to the compounds in **table SI-1**.

Table SI-1. Spectrometric and spectroscopic data of phenolic compounds in extracts as determined by UHPLC-PDA coupled to ESI-ITMS.

			[M−H]⁻			[M+H]⁺			
**Peak no.**	**T_R (min)**	**λ_{max} (nm)**	***m/z***	**CID MS² fragments (*r.a.*)[a]**	**CID MS³ fragments[b] (*r.a.*)**	***m/z***	**CID MS² fragments (*r.a.*)**	**CID MS³ fragments (*r.a.*)**	**Tentatively identified compound**
**1**	7.27	322, 398	353	191	127, 85 (95), 173 (84), 93 (60), 111 (44), 171 (27), 87 (21), 109 (20)	355	163	-	Caffeoyl quinic acid[1-3]
**2**	7.64	286	325	163	119	-	-	-	Coumaric acid hexoside A[1]
**3**	7.82	278	325	163, 119 (67), 161 (53), 101 (13)	91	-	-	-	Coumaric acid hexoside B[1]
**4**	8.81	350	447	285	285, 241 (45), 199(37), 175 (37), 217 (32), 151 (17), 257 (15), 201(12)	449	287	287	Luteolin 7-*O*-glucoside (Cynaroside)[2]
**5**	10.28	310	337	191	127	339	147		Coumaroyl quinic acid[4]
**6**	11.96	350	579	285, 447 (67), 284 (11)	285 ,460 (58), 543 (37), 559 (29), 463 (22), 403 (17), 515 (13), 461 (13)	581	287 , 449 (67)	287 , 153 (13)	Luteolin 7-*O*-apiosylglucoside[3, 5-7]
**7**	13.24	266, 338	563	269 , 431 (18)	269, 225 (22)	565	271 , 433 (73)	271	Apigenin 7-*O*-apiosylglucoside (Apiin)[3, 5-7]
**8**	13.56	346	665	621	489 , 285 (60), 579 (22), 284 (10)	667	287 , 535 (85)	287 , 258 (11)	Luteolin 7-*O*-malonyl apiosylglucoside[3, 5-7]
**9**	13.67	346	593	299 , 284 (23), 461 (12)	284	595	301 , 463 (50)	286 , 301 (24)	Chrysoeriol 7-*O*-apiosylglucoside A[3, 5-7]
**10**	13.94	254, 346	593	299 , 284 (17)	284	595	301 , 463 (51)	286 , 301 (20)	Chrysoeriol 7-*O*-apiosylglucoside B[3, 5-7]
**11**	14.87	266, 338	649	605	545, 563 (37)	651	519, 271 (80)	271	Apigenin 7-*O*-malonyl apiosylglucoside (Malonylapiin)[3, 5-7]
**12**	15.20	346	679	635	575 , 593 (50), 299 (28), 473 (16)	681	301 , 549 (80), 286 (10)	286 , 301 (24)	Chrysoeriol 7-*O*-malonyl apiosylglucoside A[3, 5-8]
**13**	15.38	346	679	635	299 , 593 (58), 284 (29), 575 (14), 503 (13)	681	301 , 549 (58), 595 (12)	286 , 301 (21)	Chrysoeriol 7-*O*-malonyl apiosylglucoside B[3, 5-8]

**14**	16.81	266, 338	605	563, 545 (57)	269, 431 (20)	607	271, 475 (64)	271	Apigenin 7-*O* acetyl apiosylglucoside (Acetylapiin)[3, 5-7]
**15**	17.17	346	635	299 , 593 (55), 284 (27), 503 (14), 575 (13)	284	637	301 , 505 (74)	286 , 301 (20)	Chrysoeriol 7-*O*-apiosylglucoside[3, 5-7]
**16**	17.95	282	557	513	349 , 469 (40), 163 (30)	-	-	-	Malonyl coumaroyl feruloyl tartaric acid A[9]
**17**	18.32	282	557	513	349 , 469 (40), 163 (30), 325 (12)	-	-	-	Malonyl coumaroyl feruloyl tartaric acid B[9]
**18**	24.56	418	307	187, 143 (30)	143	-	-	-	Bisdemethoxycurcumin [10]
**19**	25.57	422	337	217, 187 (24), 173 (21), 143 (11)	173	-	-	-	Demethoxycurcumin [10]
**20**	26.15	426	367	217, 173 (33)	173	-	-	-	Curcumin[10]

CID, collision-induced dissociation; HCD, higher energy collisional dissociation; [a]r.a., relative abundance. The threshold for fragments was ≥10%. The most intense fragment is underlined; [b] The parent ion of the MS^3 fragmentation is the fragment with 100% relative abundance in MS^2 fragmentation.

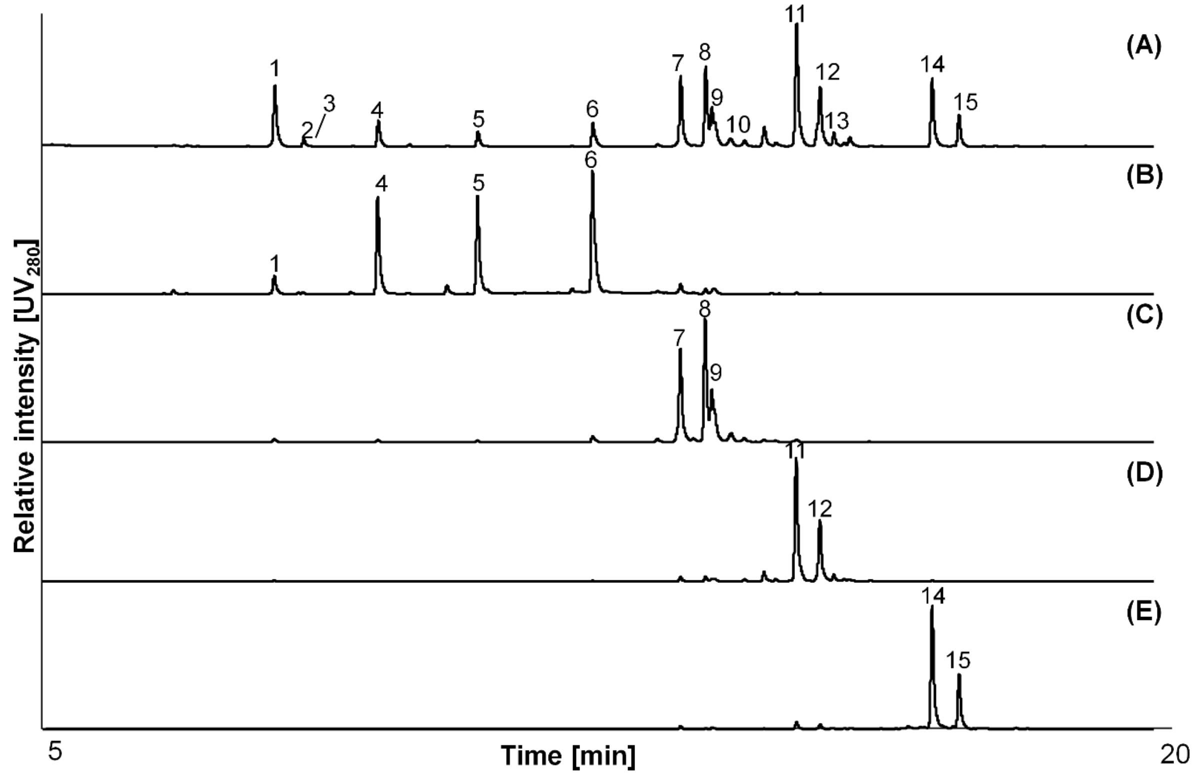

Figure SI-2. RP-UHPLC-UV profile (280 nm) of the celery extract (**A**), and pools derived from flash chromatographic separation; Pool 1 (**B**), Pool 2 (**C**), Pool 3 (**D**), and Pool 4 (**E**). Peak numbers refer to the compounds in **table SI-1**.

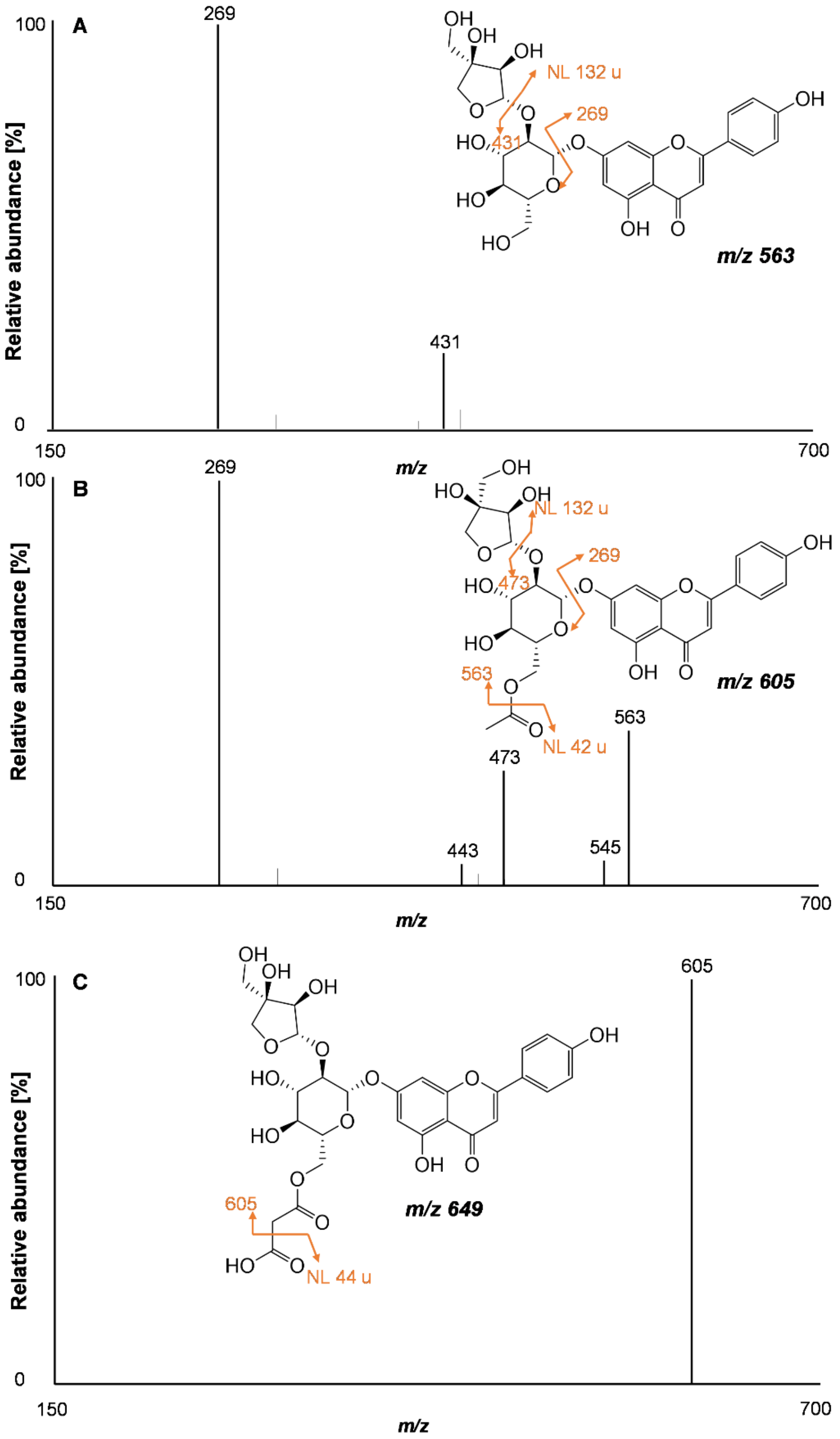

Figure SI-3. Negative mode CID MS^2 fragmentation spectra of (**A**) apigenin 7-*O*-apiosylglucoside; (**B**) apigenin 7-*O*-(6″-*O*-acetyl)-apiosylglucoside; and (**C**) apigenin 7-*O*-(6″-*O*-malonyl)-apiosylglucoside. The cut-off value for fragment labelling was 10% of the relative abundance.

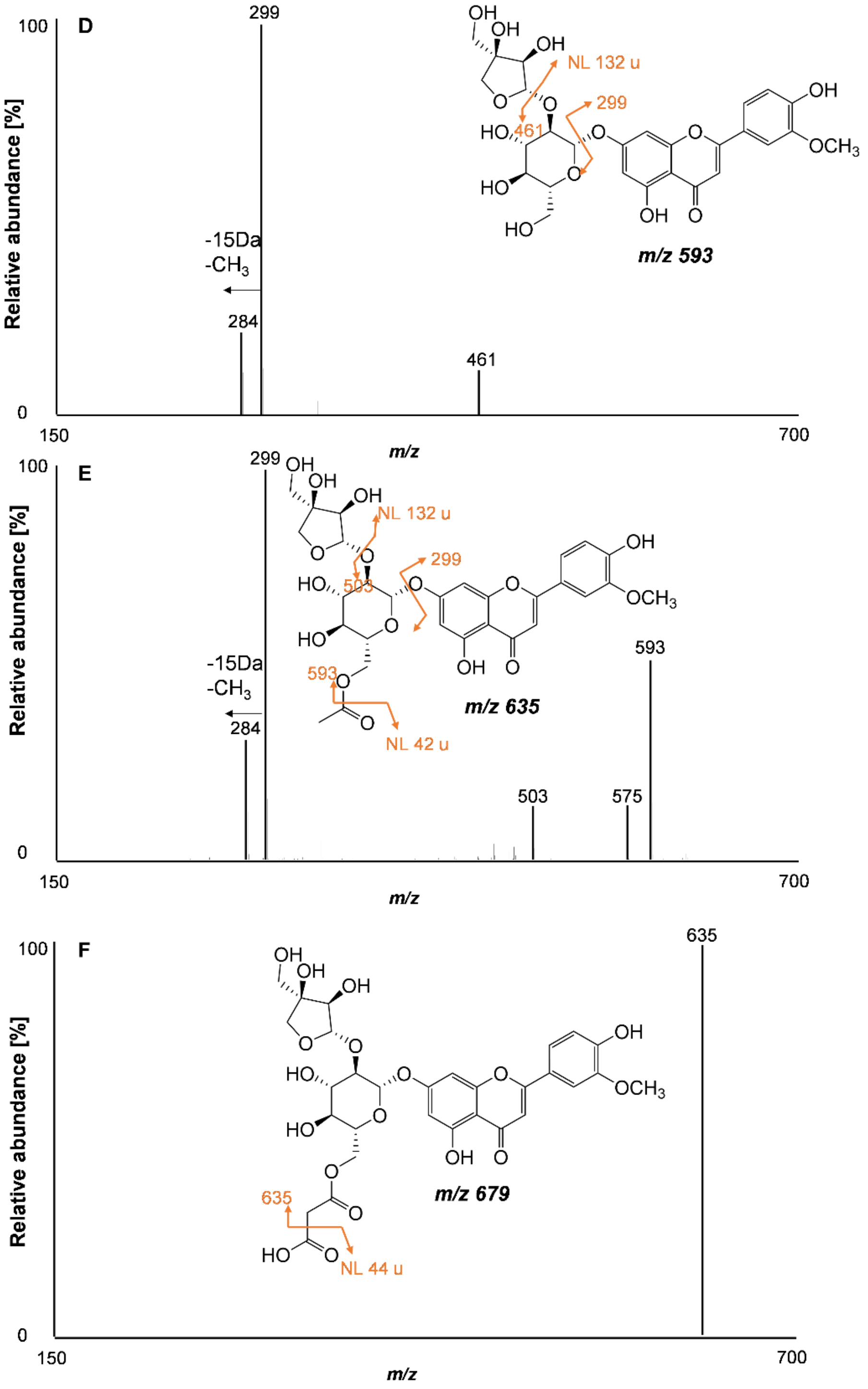

Figure SI-3 continued. Negative mode CID MS^2 fragmentation spectra of (**D**) chrysoeriol 7-*O*-apiosylglucoside; (**E**) chrysoeriol 7-*O*-(6″-*O*-acetyl)-apiosylglucoside; and (**F**) chrysoeriol 7-*O*-(6″-*O*-malonyl)-apiosylglucoside. The cut-off value for fragment labelling was 10% of the relative abundance.

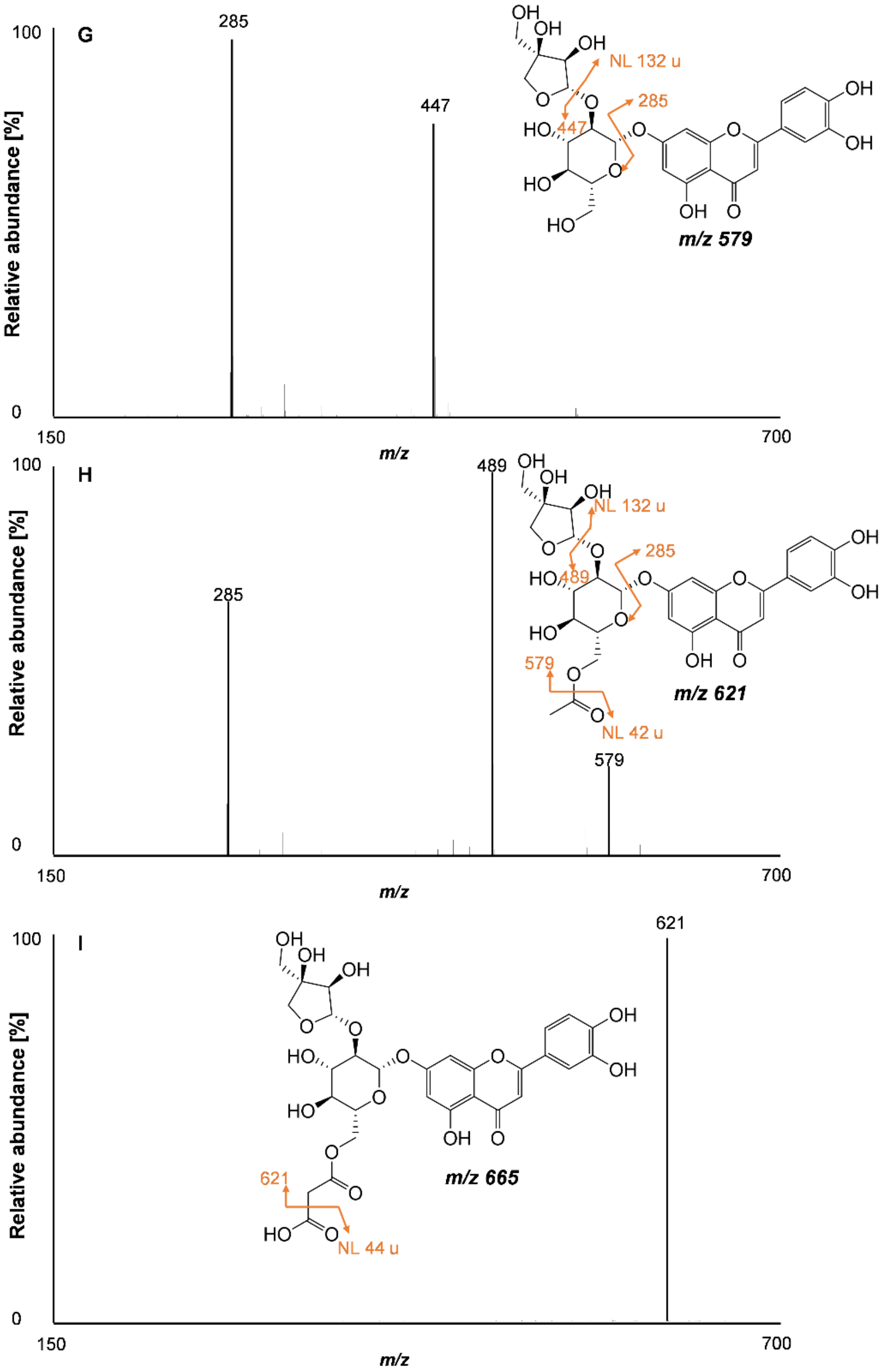

Figure SI-3 continued. Negative mode CID MS^2 fragmentation spectra of (**G**) luteolin 7-*O*-apiosylglucoside; (**H**) luteolin 7-*O*-(6′′-*O*-acetyl)-apiosylglucoside and (**I**) luteolin 7-*O*-(6′′-*O*-malonyl)-apiosylglucoside. The cut-off value for fragment labelling was 10% of the relative abundance.

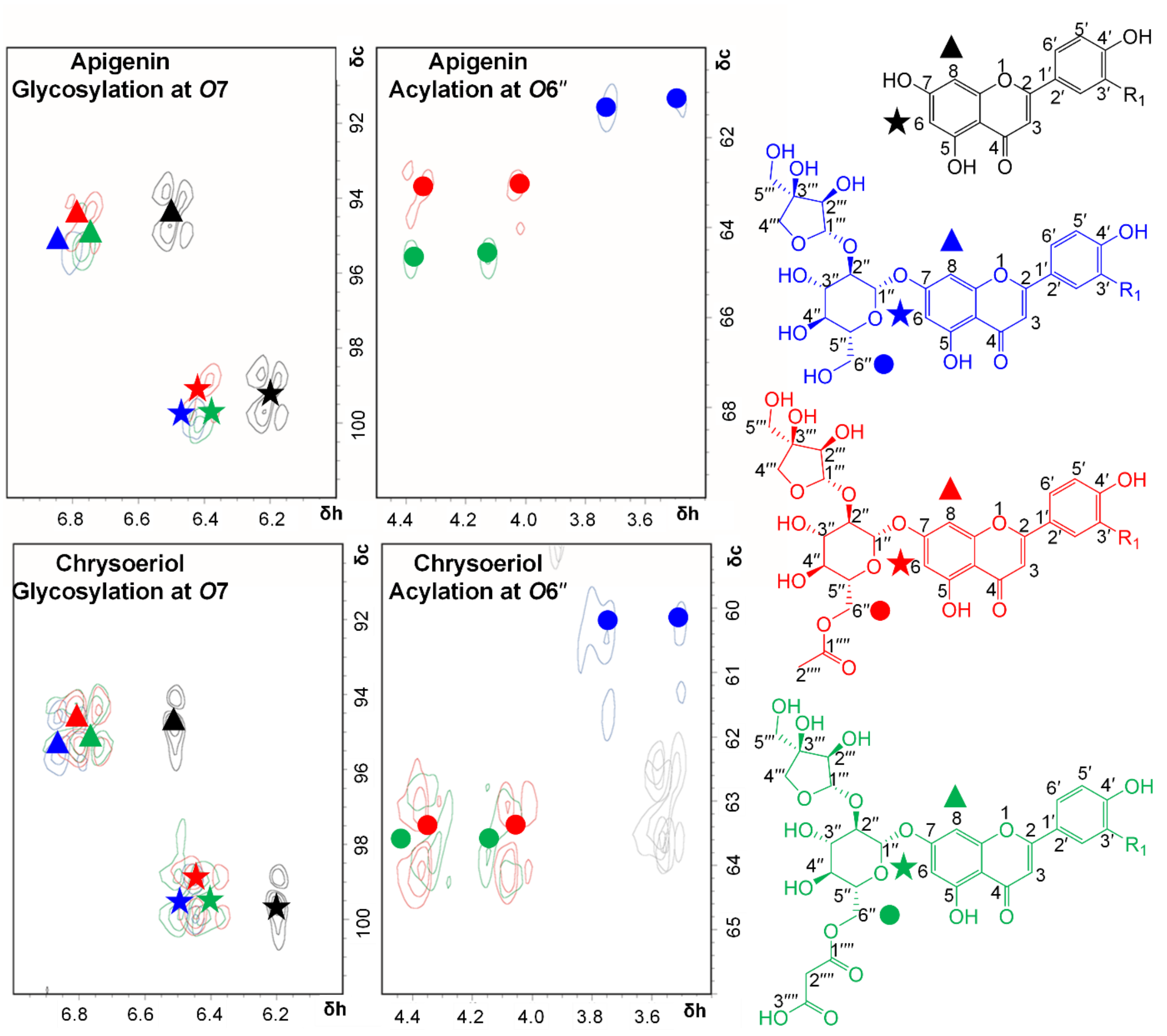

Figure SI-4. Overlayed 2D HSQC NMR spectra of the C_6-H_6 (star) and C_8-H_8 (triangle) region (δc 90-102, δh 6.0-7.0) and the $C_{6''}$-$H_{6''}$ (circle) region (δc 58-70, δh 3.4-4.5) of the apigenin and chrysoeriol aglycone (black), apiosylglucoside (blue), acetyl apiosylglucoside (red), and malonyl apiosylglucoside (green). Spectra confirm glycosylation at the *O*7 position due to the downfield shifts of C_6-H_6 and C_8-H_8, and acylation at the *O*6″ position due to the downfield shift of $C_{6''}$-$H_{6''}$.

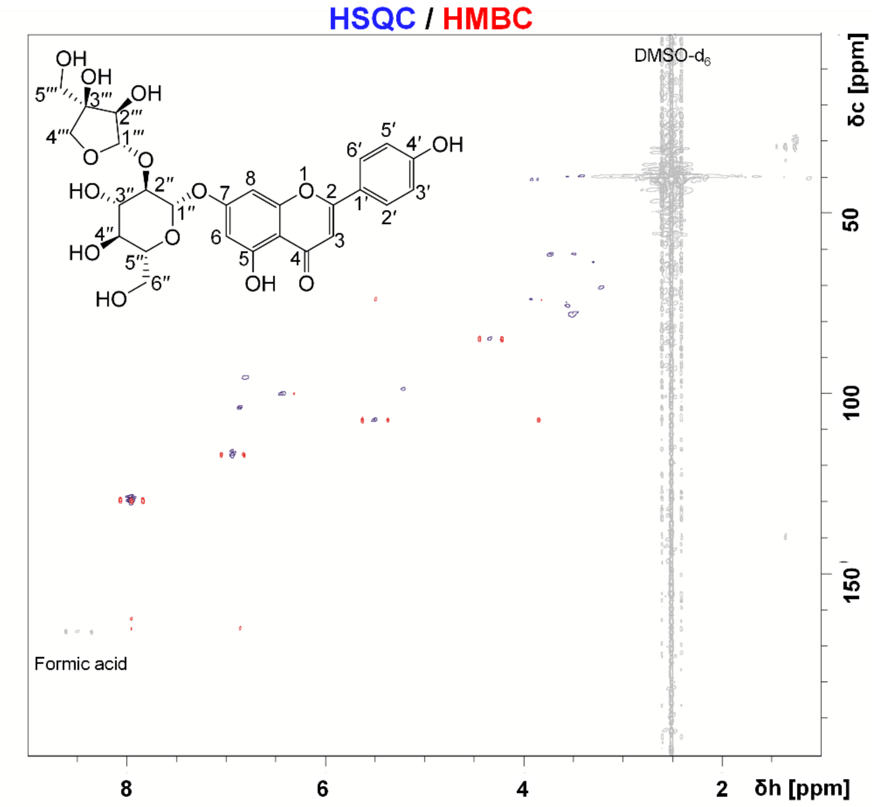

Apigenin 7-*O*-apiosylglucoside		
**Position**	**δc**	**δh, mult. (J)**
**3**	104.2	6.86, s
**4**	181.9	
**5**	161.7	
**6**	100.0	6.43, d (2.0)
**7**	162.7	
**8**	95.4	6.80, d (2.0)
**2′**	129.3	7.97, dd (8.8, 2.3)
**3′**	116.6	6.94, dd (8.8, 2.1)
**4′**	161.7	
**5′**	116.6	6.94, dd (8.8, 2.1)
**6′**	129.3	7.96, dd (8.8, 2.3)
**1″**	98.7	5.21, d (7.5)
**2″**	75.4	3.55, m
**3″**	76.1	3.56, m
**6a″**	61.4	3.49, m
**6a″**	61.4	3.72, m
**1‴**	106.2	5.49, bs
**4a‴**	73.9	3.66, m
**4b‴**	73.9	3.91, d (9.4)
**5‴**	63.6	3.29, m

Figure SI-5. 2D HSQC (blue) and HMBC (red) NMR spectra and peak annotations of apigenin 7-*O*-apiosylglucoside (apiin) measured in DMSO-d$_6$. The signal assignment of the ^{1}H NMR spectra matches with the chemical shifts of apiin reported by Eckey-Kaltenbach and co-authors.[11] The HSQC and HMBC correlations also confirmed the structure of apiin. Grey correlations are unassigned signals originating from the solvent or impurities.

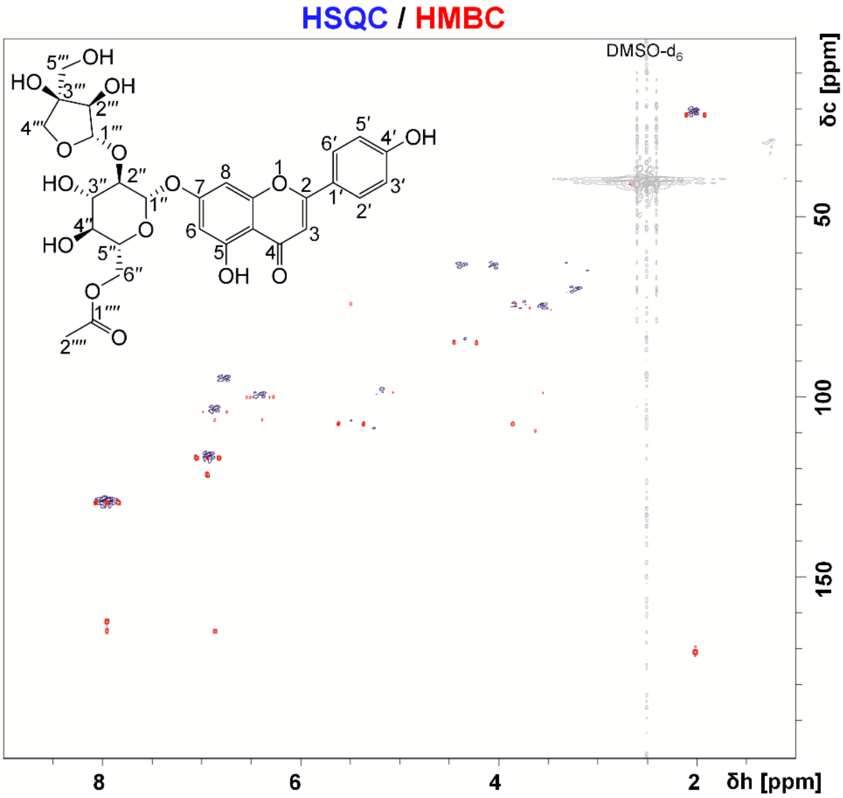

Apigenin 7-*O*-(6″-*O*-acetyl)-apiosylglucoside		
**Position**	**δc**	**δh, mult. (J)**
**3**	103.1	6.86, s
**4**	181.9	
**5**	161.4	
**6**	99.5	6.42, d (2.0)
**7**	162.4	
**8**	94.0	6.79, d (2.0)
**2′**	128.5	7.96, dd (2.2, 8.8)
**3′**	115.7	6.94, dd (2.2, 8.8)
**4′**	161.4	
**5′**	115.7	6.94, dd (2.2, 8.8)
**6′**	128.5	7.96, dd (2.2, 8.8)
**1″**	97.3	5.19, d (2.8)
**2″**	75.6	3.53, m
**3″**	76.1	3.56, m
**6a″**	63.3	4.05, m
**6a″**	63.3	4.35, m
**1‴**	108.7	5.26, bs
**2‴**	76.7	3.75, bs
**4a‴**	73.9	3.56, d (9.4)
**4b‴**	73.9	3.84, d (9.4)
**5‴**	64.2	
**1⁗**	170.6	
**2⁗**	20.4	2.01, d (2.4)

Figure SI-6. 2D HSQC (blue) and HMBC (red) NMR spectra and peak annotations of apigenin 7-*O*-(6′′-*O*-acetyl)-apiosylglucoside (6′′-acetylapiin) measured in DMSO-d$_6$. The signal assignment of the ^{1}H and ^{13}C NMR spectra matches with the chemical shifts of 6′′-acetylapiin reported by Yoshikawa et al.[12] The HSQC and HMBC correlations also confirmed the structure of acetylapiin. Grey correlations are unassigned signals originating from the solvent or impurities.

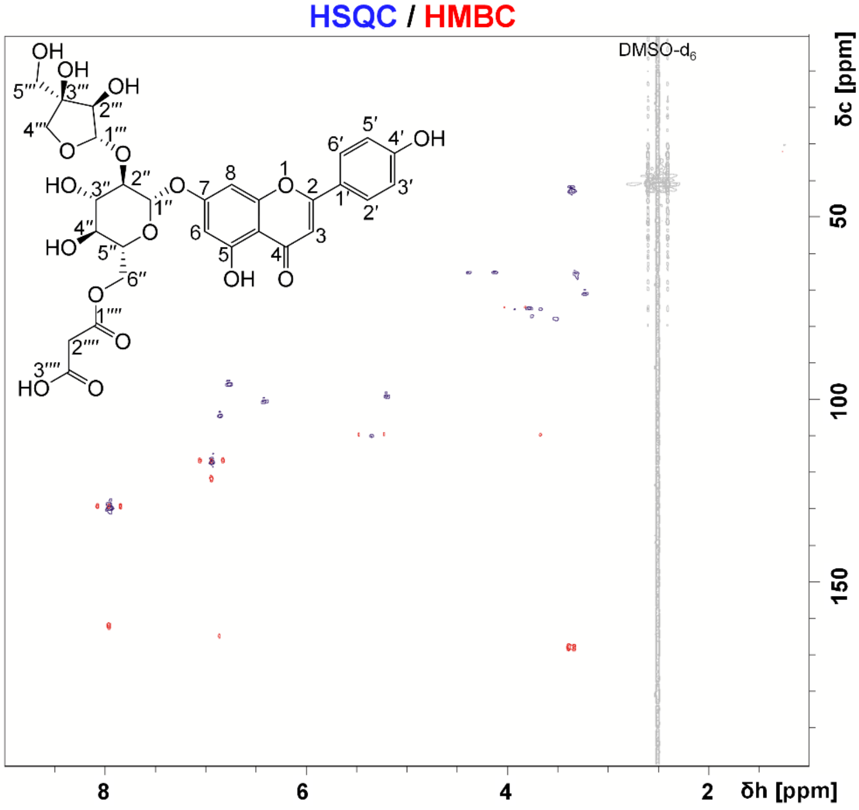

Apigenin 7-*O*-(6″-*O*-malonyl)-apiosylglucoside		
**Position**	**δc**	**δh, mult. (J)**
**3**	103.1	6.86, s
**4**	181.9	
**5**	161.4	
**6**	99.3	6.39, d (2.0)
**7**	162.7	
**8**	94.8	6.79, d (2.0)
**2′**	129.2	7.96, dd (2.2, 8.8)
**3′**	116.5	6.94, dd (2.2, 8.8)
**4′**	161.5	
**5′**	117.2	6.94, dd (2.2, 8.8)
**6′**	121.0	7.96, dd (2.2, 8.8)
**1″**	98.6	5.19, d (2.8)
**2″**	76.2	3.53, m
**3″**	76.2	3.56, m
**4″**	74.4	3.79, m
**5″**	69.2	3.25, m
**6a″**	64.4	4.13, m
**6a″**	64.4	4.39, m
**1‴**	109.2	5.32, bs
**2‴**	76.5	3.75, bs
**4a‴**	74.7	3.68, d (9.4)
**4b‴**	74.7	3.93, d (9.4)
**5‴**	70.7	3.23, m
**1⁗/3⁗**	167.5	
**2⁗**	42.0	3.36, m

Figure SI-7. 2D HSQC (blue) and HMBC (red) NMR spectra and peak annotations of apigenin 7-*O*-(6″-*O*-malonyl)-apiosylglucoside (6″-malonylapiin) measured in DMSO-d$_6$. The signal assignment of the ^{1}H NMR spectra matches with the chemical shifts of 6″-malonylapiin reported by Eckey-Kaltenbach et al.[11] The HSQC and HMBC correlations also confirmed the structure of malonylapiin. Grey correlations are unassigned signals originating from the solvent or impurities.

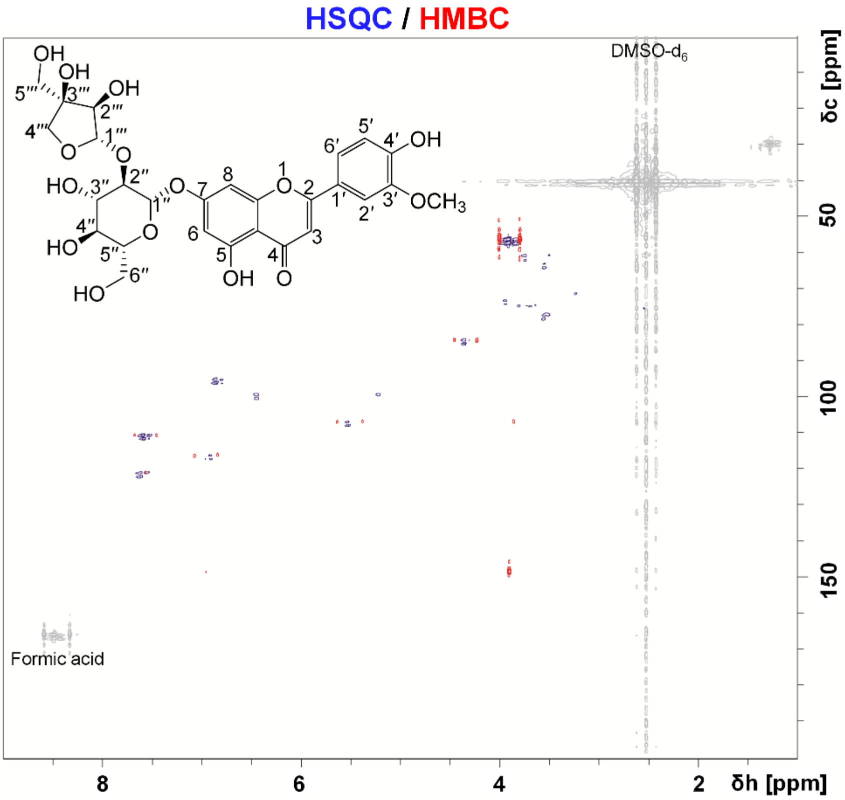

Position	δc	δh, mult. (J)
3	115.7	6.90, s
6	99.5	6.43, d (2.0)
8	94.6	6.82, d (2.0)
2′	111.0	7.59, m
4′	148.9	
5′	115.7	6.94, d (8.4)
6′	121.7	7.62, dd (2.0, 8.9)
3′-OCH_3	56.0	3.89, s
1″	98.8	5.22, d (7.5)
2″	77.9	3.55, m
3″	77.0	3.53, m
4″	77.0	3.84,m
5″	70.7	3.23, m
6a″	60.4	3.49,m
6b″	60.4	3.74, m
1‴	106.9	5.50, bs
4a‴	73.5	3.85, d (9.0)
4b‴	73.8	3.91, d (9.2)
5″	70.2	3.23, m

Chrysoeriol 7-*O*-apiosylglucoside

Figure SI-8. 2D HSQC (blue) and HMBC (red) NMR spectra and peak annotations of chrysoeriol 7-*O*-apiosylglucoside measured in DMSO-d6. The signals for the phenolic backbone of this compound were similar to chrysoeriol as reported by Park et al.[13] Different from the signals reported by Park et al., *H*6 and *H*8 showed an downfield shift which is due to the presence of the apiosylglycoside on the *O*7 position similarly as what was seen for compound A compared to the apigenin aglycon. Presence of the methoxy group at *C*3′ was evidenced by the new signals at δ3.89 (s) in H and δ56 in C and the HMBC and HSQC spectra.[14] Grey correlations are unassigned signals originating from the solvent or impurities.

Supporting Information with "Interactions of natural flavones with iron are affected by 7-*O*-glycosylation, but not by additional 6″-*O*-acylation" by Bijlsma, de Bruijn, Koppelaar, Sanders, Velikov, and Vincken.

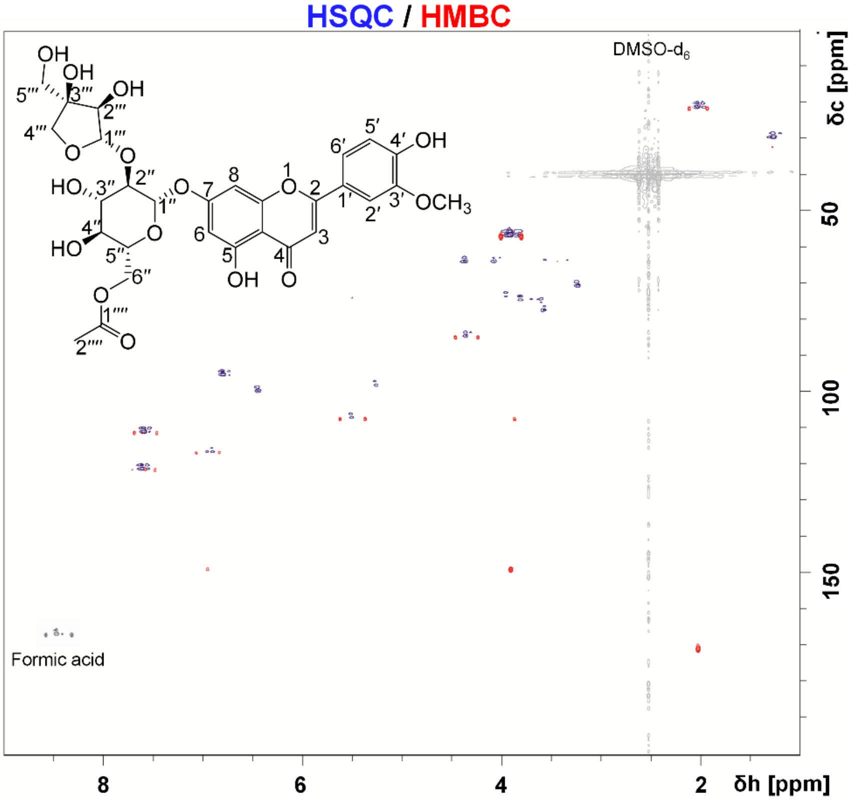

Chrysoeriol 7-*O*-(6″-*O*-acetyl)-apiosylglucoside		
Position	δc	δh, mult. (J)
3	115.8	6.90, s
6	99.3	6.43, d (2.0)
8	94.7	6.79, d (2.0)
2′	110.6	7.57, m
4′	149.0	
5′	116.0	6.96, d (8.8)
6′	121.0	7.59, dd (2.0, 8.9)
3′-OCH_3	56.9	3.89, s
1″	98.2	5.26, d (7.4)
2″	77.5	3.58, m
3″	74.5	3.62, m
4″	74.5	3.80, m
5″	70.4	3.27, m
6a″	64.0	4.07, m
6n″	64.0	4.36, m
1‴	106.9	5.50, bs
4a‴	73.7	3.85, d (9.7)
4b‴	73.7	3.95, d (10.0)
5‴	70.6	3.27, m
1⁗	107.9	
2⁗	21.2	2.03, s

Figure SI-9. 2D HSQC (blue) and HMBC (red) NMR spectra and peak annotations of chrysoeriol 7-*O*-(6″-*O*-acetyl)-apiosylglucoside measured in DMSO-d6. The signals for the phenolic backbone of this compound were similar to chrysoeriol as reported by Park et al.[13] Different from the signals reported by Park et al., *H*6 and *H*8 showed an downfield shift which is due to the presence of the apioglycoside on the *O*7 position. Presence of the methoxy group at *C*3′ was evidenced by the new signals at δ3.89 (s) in H and δ56 in C and the HMBC and HSQC spectra.[14] This compound showed similar signals as compound G which was identified as a chrysoeriol 7-*O*-apiosylglucoside but the diagnostic differences of 42 in RP-UHPLC-MS indicated the presence of an acetyl substitution. Presence of the acetyl was evidenced by the new signal for the 2⁗ position at δ2.03 in H and δ21.2 in C for acetyl. Grey correlations are unassigned signals originating from the solvent or impurities.

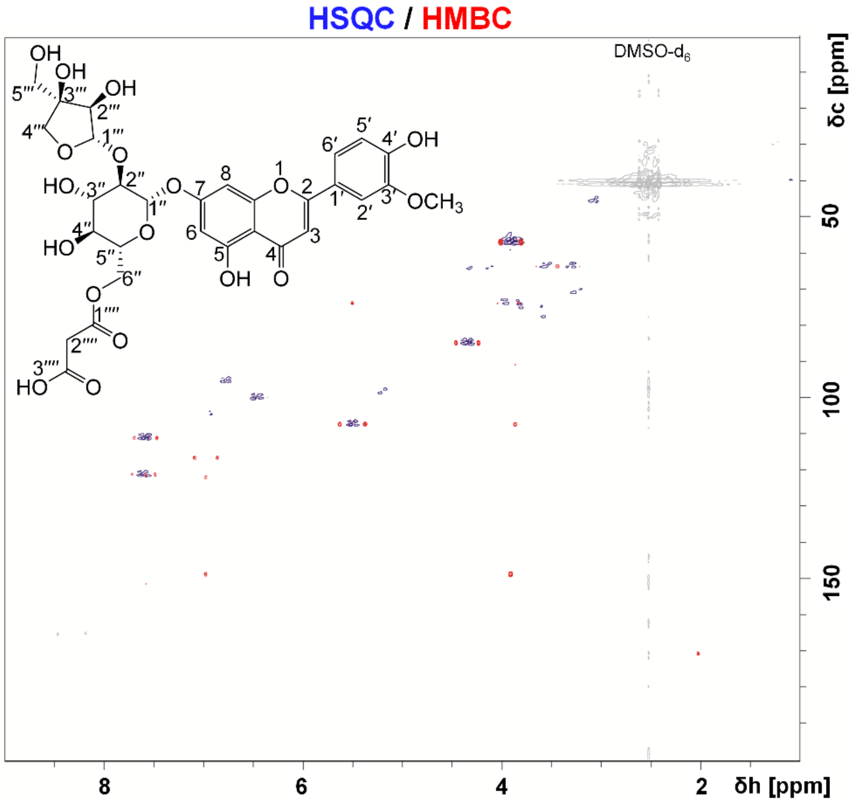

Chrysoeriol 7-*O*-(6″-*O*-malonyl)-apiosylglucoside		
Position	δc	δh, mult. (J)
3	115.7	6.88, s
6	99.3	6.43, d (2.1)
8	94.4	6.78, d (2.0)
2′	111.0	7.58, m
4′	148.6	
5′	116.4	6.96, d (8.8)
6′	121.0	7.60, dd (2.0, 8.8)
3′-OCH_3	56.4	3.89, s
1″	97.9	5.20, d (7.2)
2″	77.3	3.58, m
3″	74.5	3.60, m
4″	74.6	3.77, m
5″	70.4	3.28, m
6a″	63.7	4.11, m
6b″	63.7	4.33, m
1‴	106.9	5.50, bs
4a‴	73.7	3.85, d (9.4)
4b‴	73.7	3.93, d (9.4)
5‴	70.6	3.27, m
1″‴/3″‴	147.7	
2″‴	44.6	3.07, m

Figure SI-10. 2D HSQC (blue) and HMBC (red) NMR spectra and peak annotations of chrysoeriol 7-*O*-(6″-*O*-malonyl)-apiosylglucoside measured in DMSO-d$_6$. The signals for the phenolic backbone of this compound were similar to chrysoeriol as reported by Park et al.[13] Different from the signals reported by Park et al., *H*6 and *H*8 showed an downfield shift which is due to the presence of the apioglycoside on the *O*7 position. Presence of the methoxy group at *C*3′ was evidenced by the new signals at δ3.89 (s) in H and δ56 in C and the HMBC and HSQC spectra.[14] This compound showed similar signals as compound G which was identified as a chrysoeriol 7-*O*-apiosylglucoside but the diagnostic differences of 86 in RP-UHPLC-MS indicated the presence of an malonyl substitution. Presence of the malonyl was evidenced by the new signal for the 2″‴ position at δ3.07 in H and δ44.6 in C. Grey correlations are unassigned signals originating from the solvent or impurities.

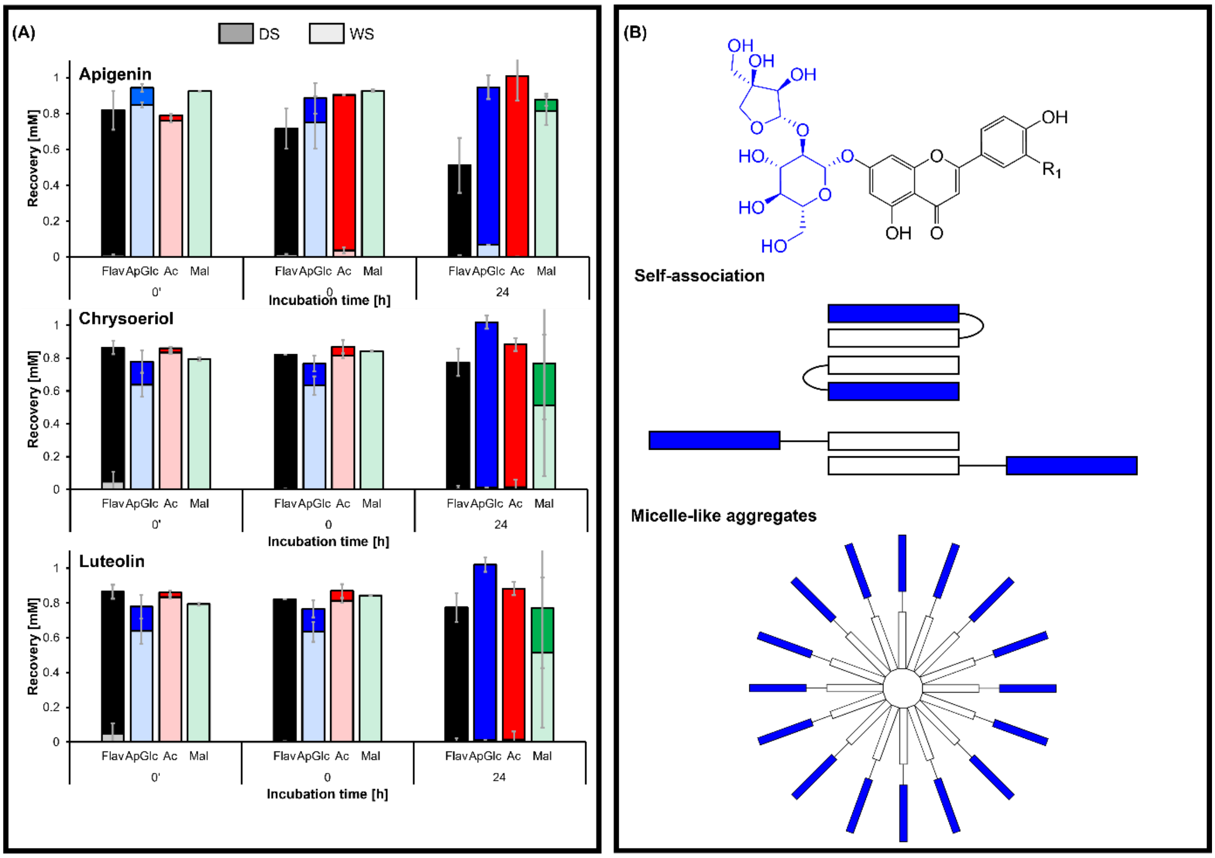

Fig. SI-11. (A) Recovery of the flavone aglycons (Flav; black) and apiosylglucoside (ApGlc; blue) with additional acetylation (Ac; red) or malonylation (Mal; green) in the WS and DS fractions in absence of $FeSO_4$. Time points shown are before the adjustment of the pH ($t_{0'}$) and after 0 or 24 h of incubation at pH 6.5 in an aqueous solution. Error bars indicate the standard deviation of independent duplicates. Significance (Tukey's test, $p < 0.05$) of differences in the total recovery are indicated in Table SI-2. (**B**) Proposed self-association or micelle-like aggregate formation of flavone glycosides, the hydrophilic moieties are indicated in blue and hydrophobic moieties in white.

Supporting Information with "Interactions of natural flavones with iron are affected by 7-*O*-glycosylation, but not by additional 6″-*O*-acylation" by Bijlsma, de Bruijn, Koppelaar, Sanders, Velikov, and Vincken.

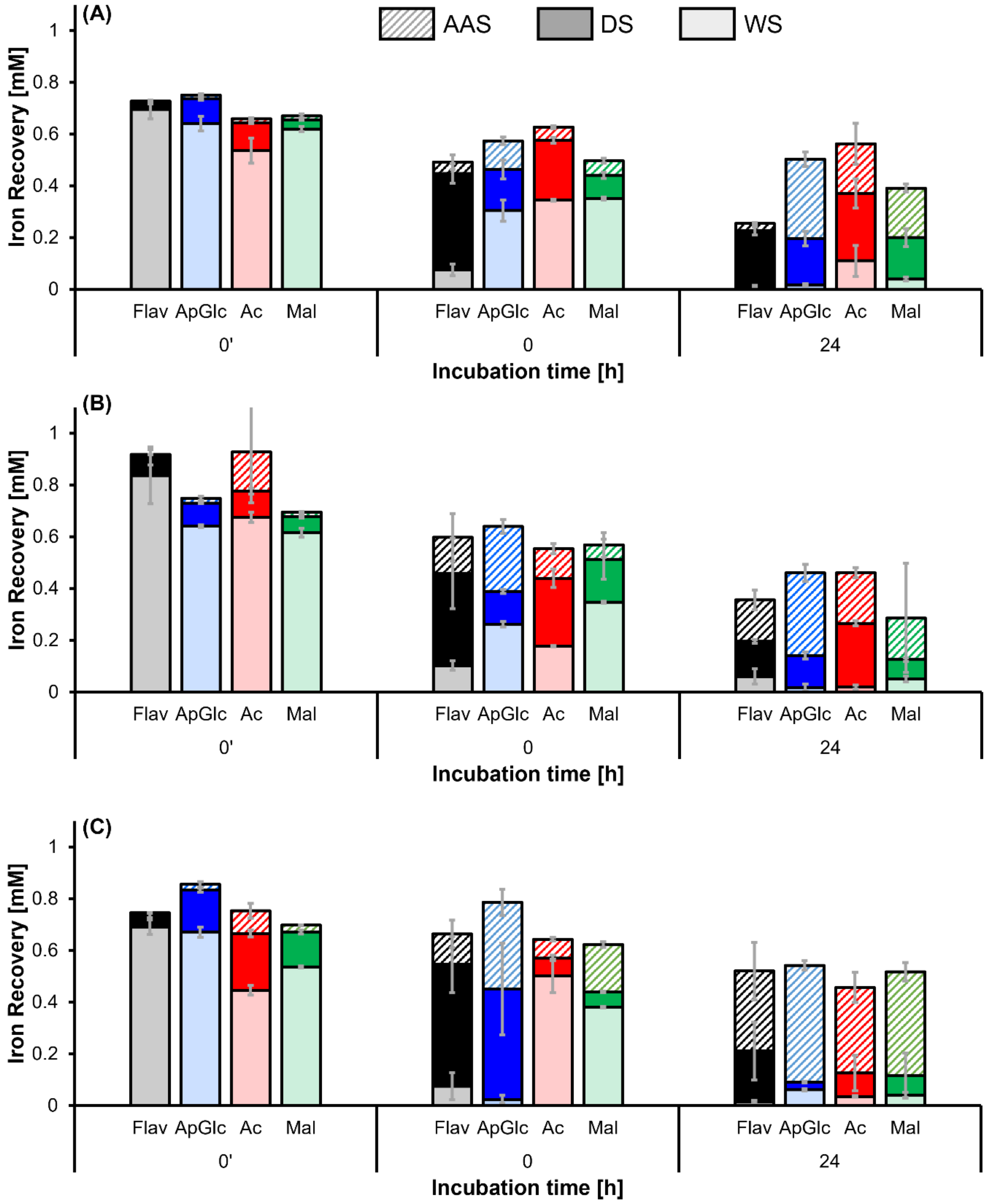

Fig. SI-12. Recovery of iron in presence of equimolar concentration of the flavone aglycons (Flav; black) and apiosylglucoside (ApGlc; blue) with additional acetylation (Ac; red) or malonylation (Mal; green) for (**A**) apigenin, (**B**) chrysoeriol, and (**C**) luteolin in the WS, DS, and AAS fractions. Time points shown are before the adjustment of the pH ($t_{0'}$) and after 0 or 24 h of incubation at pH 6.5 in an aqueous solution. Error bars indicate the standard deviation of independent duplicates. Significance (Tukey's test, $p < 0.05$) of differences in the total recovery are indicated in Table SI-2.

Table SI-2. Statistical analysis of the flavone and iron recovery in total and for the recovery in each of the three different fractions: water soluble (WS), DMSO soluble (DS), and ascorbic acid soluble (AAS) fractions. The quantitative differences were assessed by Tukey's *post hoc* comparison ($p < 0.05$). Significant differences in recovery compared to the other time points for the same flavone and of the three individual fractions (*i.e.,* WS, DS, and AAS) are indicated with a different letter.

		Flavone recovery in presence of $FeSO_4$				Flavone recovery in absence of $FeSO_4$				Iron recovery in presence of flavone			
**Flavone**	**Time**	**Total**	**WS**	**DS**	**AAS**	**Total**	**WS**	**DS**	**AAS**	**Total**	**WS**	**DS**	**AAS**
	0'	a	a	a	a	a	a	a	n.a.	a	a	a	a
**apigenin**	0	ab	a	a	a	a	a	a	n.a.	b	b	b	a
	24	b	a	b	a	a	a	a	n.a.	c	b	c	a
**apigenin 7-*O*-apiosylglucoside**	0'	a	a	a	a	a	a	a	n.a.	a	a	a	a
	0	a	b	a	a	a	a	a	n.a.	b	b	a	b
	24	a	b	a	b	a	b	b	n.a.	b	c	a	c
**apigenin 7-*O*-(6″-*O*-acetyl)-apiosylglucoside**	0'	a	a	a	a	a	a	a	n.a.	a	a	a	a
	0	a	a	a	a	a	b	b	n.a.	a	b	a	a
	24	a	a	a	b	a	b	b	n.a.	a	c	a	a
**apigenin 7-*O*-(6″-*O*-malonyl)-apiosylglucoside**	0'	a	a	a	a	a	a	a	n.a.	a	a	a	a
	0	ab	b	a	a	a	a	a	n.a.	b	b	ab	a
	24	b	c	b	b	a	a	a	n.a.	c	c	b	b
	0'	a	a	a	a	a	a	a	n.a.	a	a	a	a
**chrysoeriol**	0	a	a	a	a	a	a	a	n.a.	ab	b	a	a
	24	a	a	a	a	b	a	a	n.a.	b	b	a	a
**chrysoeriol 7-*O*-apiosylglucoside**	0'	a	a	a	a	a	a	a	n.a.	a	a	a	a
	0	a	a	a	a	a	a	a	n.a.	b	b	b	b
	24	a	a	a	a	a	b	b	n.a.	c	c	b	b
**chrysoeriol 7-*O*-(6″-*O*-acetyl)-apiosylglucoside**	0'	a	a	a	a	a	a	a	n.a.	a	a	a	a
	0	a	a	a	a	a	a	a	n.a.	a	b	b	a
	24	a	a	a	a	a	b	b	n.a.	a	c	b	a
**chrysoeriol 7-*O*-(6″-*O*-malonyl)-apiosylglucoside**	0'	a	a	a	a	a	a	a	n.a.	a	a	a	a
	0	ab	b	b	a	a	a	a	n.a.	a	b	a	a
	24	b	c	c	b	a	a	a	n.a.	a	c	a	a

n.a. not applicable, no ascorbic acid soluble fraction was present.

Table SI-2. Continued

		Flavone recovery in presence of $FeSO_4$				Flavone recovery in absence of $FeSO_4$				Iron recovery in presence of flavone			
**Flavone**	**Time**	**Total**	**WS**	**DS**	**AAS**	**Total**	**WS**	**DS**	**AAS**	**Total**	**WS**	**DS**	**AAS**
**luteolin**	0'	a	a	a	a	a	a	a	n.a.	a	a	a	a
	0	a	a	ab	ab	a	a	a	n.a.	a	b	b	a
	24	a	a	b	b	a	a	a	n.a.	a	b	b	a
**luteolin 7-*O*-apiosylglucoside**	0'	a	a	a	a	a	a	a	n.a.	a	a	a	a
	0	a	a	b	a	a	a	a	n.a.	ab	b	a	b
	24	a	a	b	a	a	a	a	n.a.	b	b	a	b
**luteolin 7-*O*-(6″-*O*-acetyl)-apiosylglucoside**	0'	a	a	a	a	a	a	a	n.a.	a	a	a	a
	0	a	a	b	a	a	a	a	n.a.	a	a	a	ab
	24	a	a	b	a	a	a	a	n.a.	b	b	a	b
**luteolin 7-*O*-(6″-*O*-malonyl)-apiosylglucoside**	0'	a	a	a	a	a	a	a	n.a.	a	a	a	a
	0	a	ab	b	ab	a	a	a	n.a.	ab	b	a	b
	24	a	b	b	b	a	a	a	n.a.	b	c	a	c

n.a. not applicable, no ascorbic acid soluble fraction was present.

Possible self-association of the flavones was tested using a dilution series of the (acylated) apigenin glycosides in phosphate buffer at pH 6.5. Self-associated complexes that are formed by π-π or CH-π stacking show a nonlinear relationship between the intensity of self-association induced absorbance and concentration of the flavones, because the complexes are disrupted below a certain concentration threshold (*i.e.*, <1 mM).[15] In our system, we observed a linear relationship between the concentration and absorbance at 400 nm (**Fig. SI-13**). This is an indication that the bathochromic shift of the (acylated) glycosides is not caused by self-association.

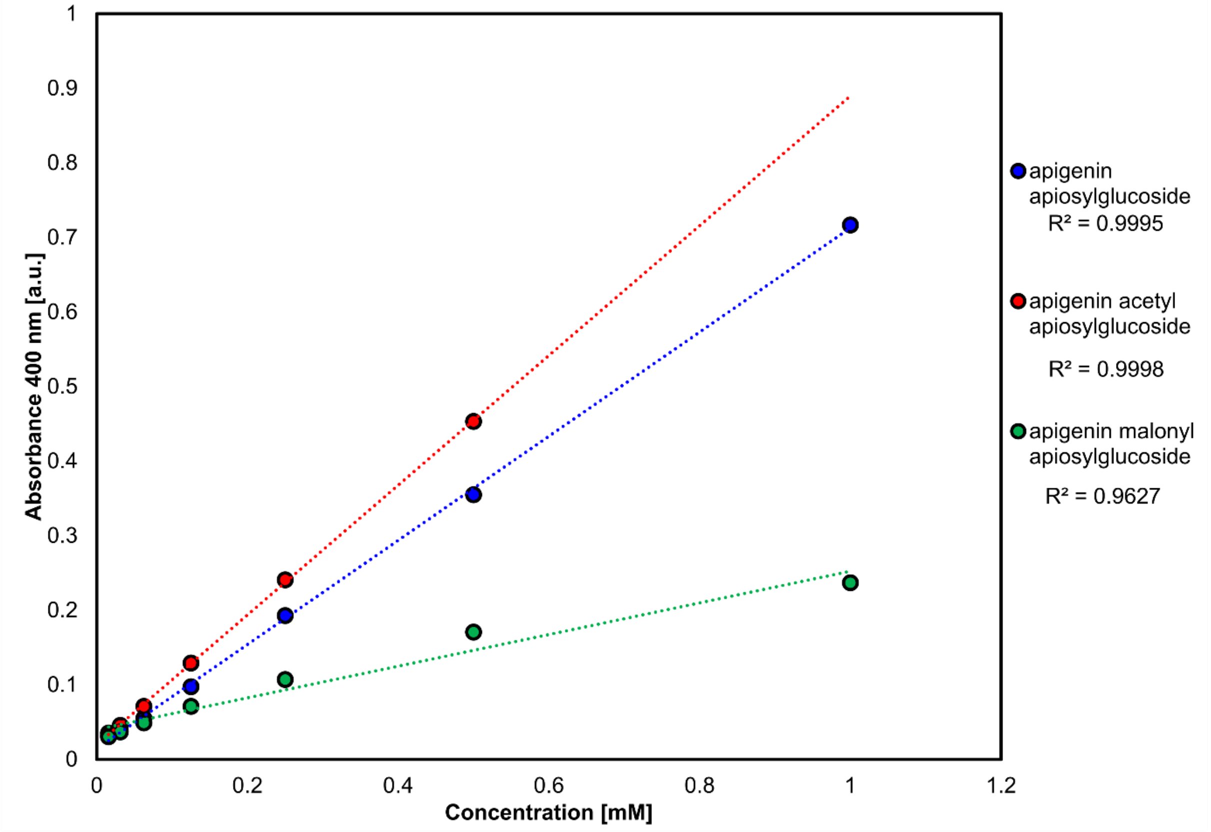

Fig. SI-13. Absorbance at 400 nm of apigenin 7-*O*-apiosylglucoside, apigenin 7-*O*-(6′′-*O*-acetyl)-apiosylglucoside, apigenin 7-*O*-(6′′-*O*-malonyl)-apiosylglucoside at different concentrations of flavone and measured in phosphate buffer at pH 6.5. For apigenin 7-*O*-(6′′-*O*-acetyl)-apiosylglucoside the point at 1 mM could not be taken into account because of precipitation at this concentration.

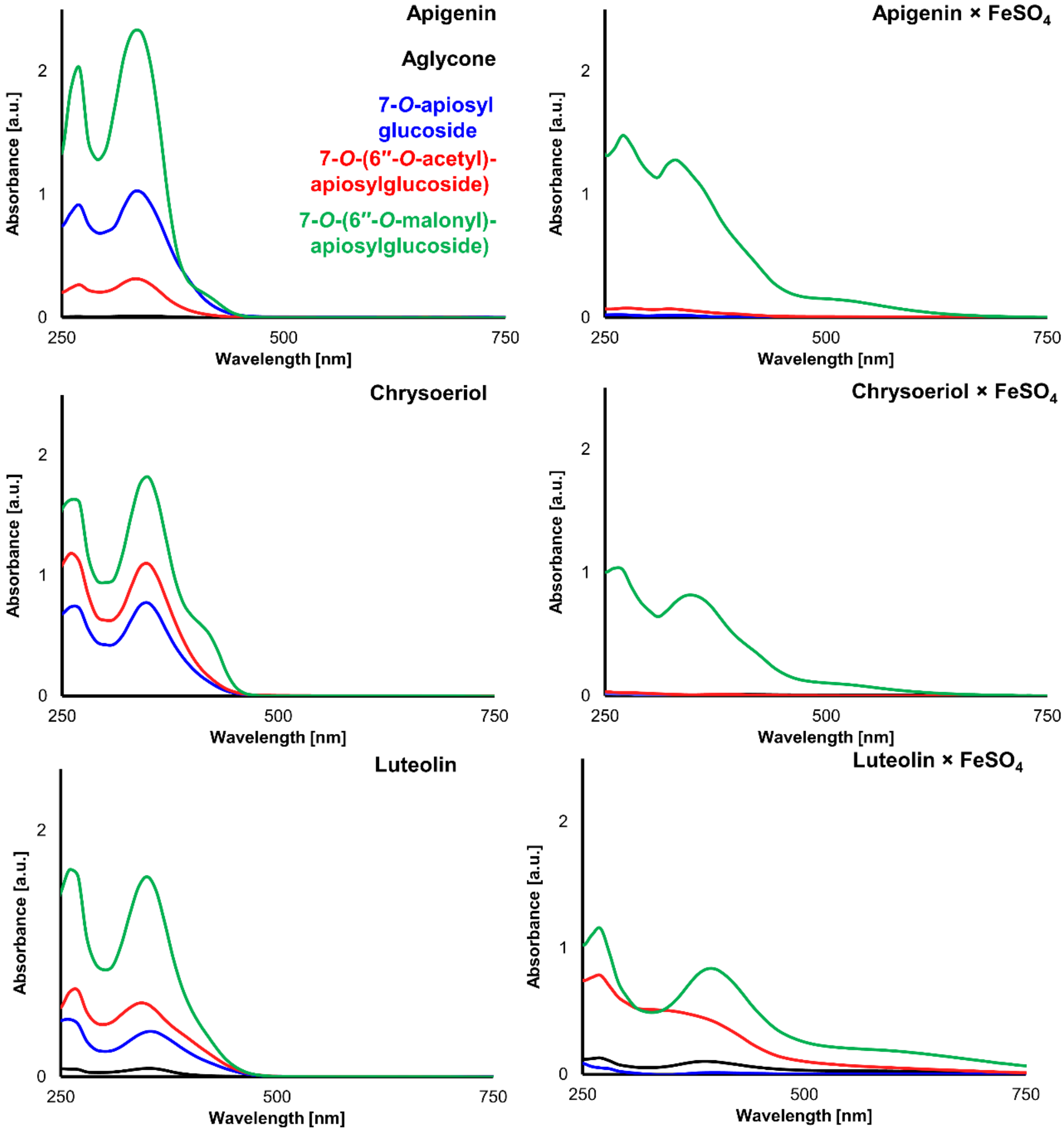

Fig. SI-14. UV-Vis absorbance spectra of the WS fraction of acylated flavone glycosides at pH 6.5 (t=0) in absence and presence of equimolar concentration $FeSO_4$.

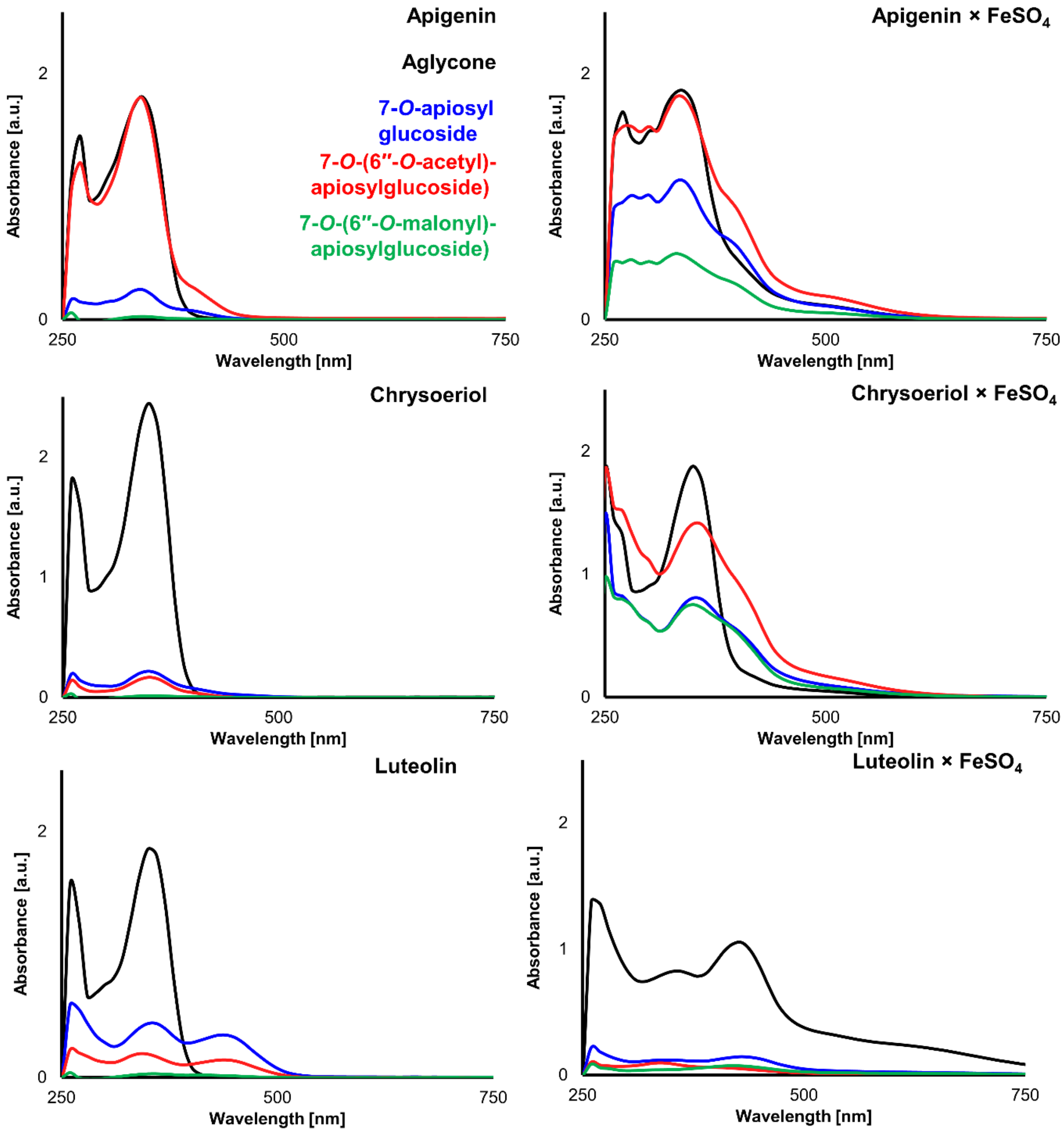

Fig. SI-15. UV-Vis absorbance spectra of the DS fraction of acylated flavone glycosides at pH 6.5 (t=0) in absence and presence of equimolar concentration $FeSO_4$.

The spectra of luteolin 7-*O*-apiosylglucoside and luteolin 7-*O*-(6″-*O*-acetyl)-apiosylglucoside in absence of iron show a shoulder band at 450 nm in the DS fraction. We suggest that the shoulder band that is observed here is probably caused by impurities that were present in the sample and not because of oxidation of the parent molecule. This is further confirmed by the fact that the new bands were also observed in the sample at t0', which was the sample before pH adjustment and incubation.

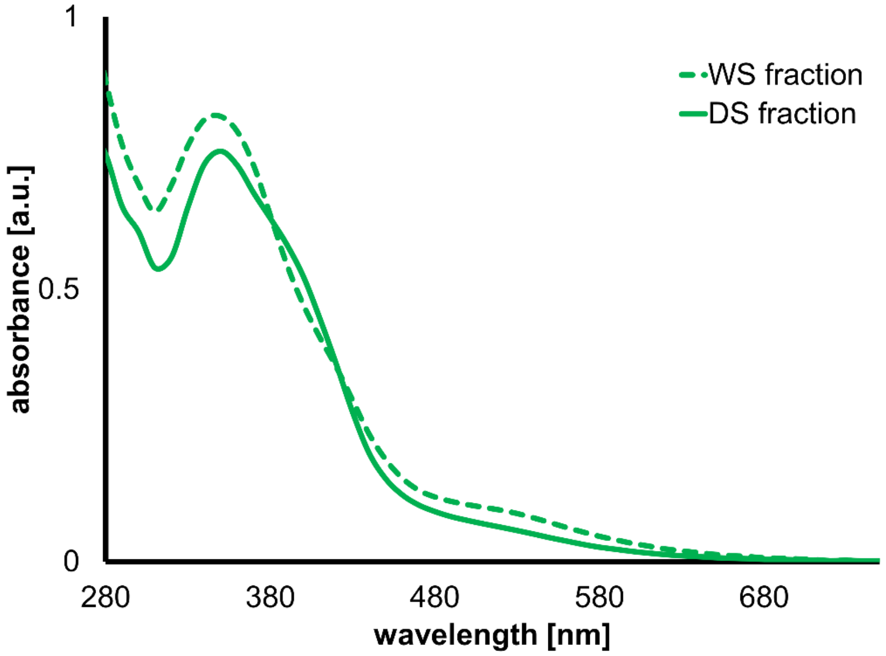

Fig. SI-16. UV-Vis absorbance spectra of the WS fraction (dashed line) and DS fraction (solid line) of chrysoeriol 7-*O*-(6′′-*O*-malonyl)-apiosylglucoside at pH 6.5 (t=0) in the presence of equimolar concentration $FeSO_4$.

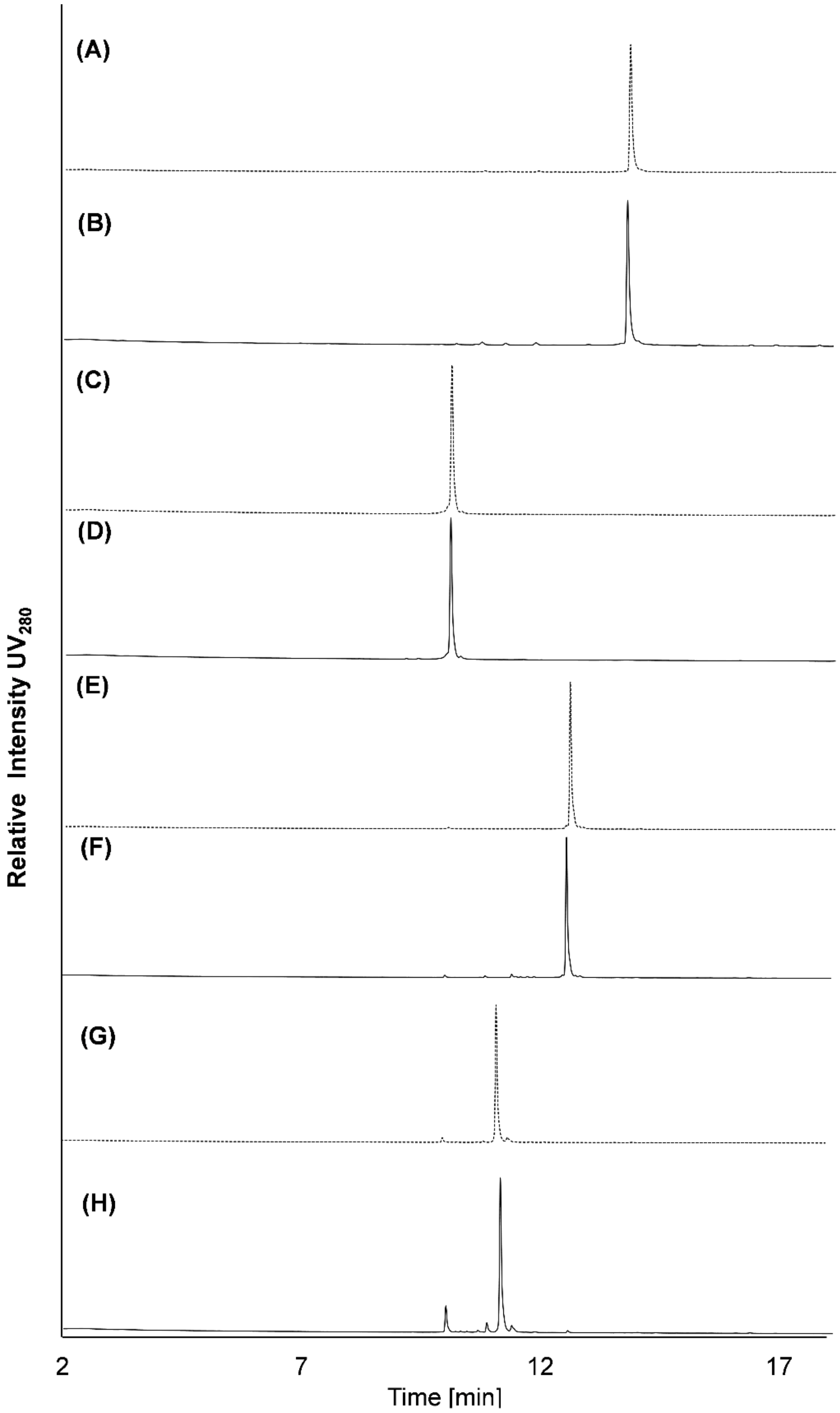

Figure SI-17. Combined RP-UHPLC-PDA (280 nm) chromatograms from the WS and DS fractions of (**A**) apigenin × $FeSO_4$ ($t_{0'}$); (**B**) apigenin × $FeSO_4$ (t_{24}); (**C**) apigenin 7-*O*-apiosylglucoside × $FeSO_4$ ($t_{0'}$); (**D**) apigenin 7-*O*-apiosylglucoside × $FeSO_4$ (t_{24}); (**E**) apigenin 7-*O*-6″-acetyl apiosylglucoside × $FeSO_4$ ($t_{0'}$); (**F**) apigenin acetyl 7-*O*-6″-apiosylglucoside × $FeSO_4$ (t_{24}); (**G**) apigenin 7-*O*-6″-malonyl apiosylglucoside × $FeSO_4$ ($t_{0'}$); and (**H**) apigenin 7-*O*-6″-malonyl apiosylglucoside × $FeSO_4$ (t_{24}).

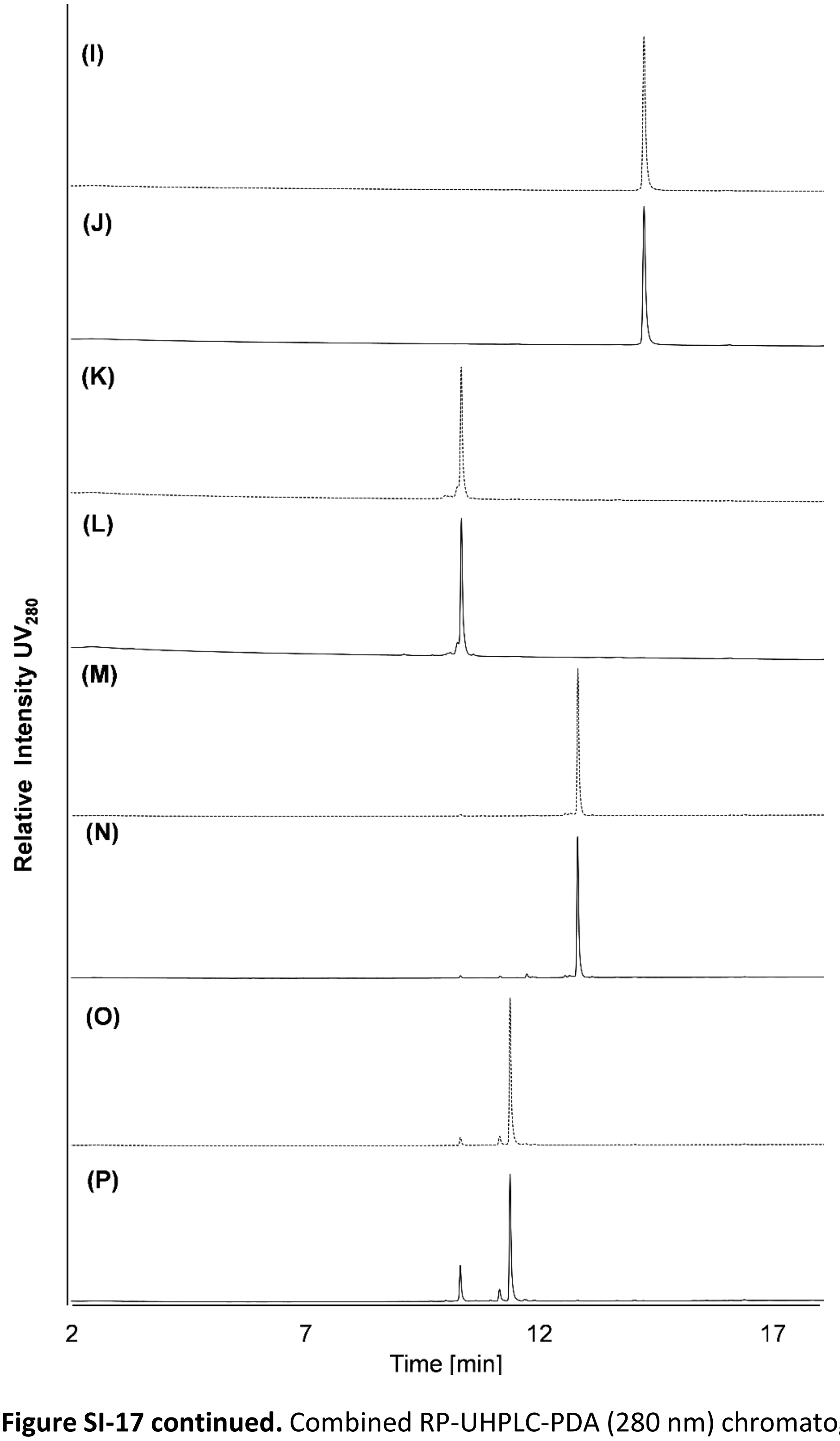

Figure SI-17 continued. Combined RP-UHPLC-PDA (280 nm) chromatograms from the WS and DS fractions of (**I**) chrysoeriol × $FeSO_4$ ($t_{0'}$); (**J**) chrysoeriol × $FeSO_4$ (t_{24}); (**K**) chrysoeriol 7-*O*-apiosylglucoside × $FeSO_4$ ($t_{0'}$); (**L**) chrysoeriol 7-*O*-apiosylglucoside × $FeSO_4$ (t_{24}); (**M**) chrysoeriol 7-*O*-6″-acetyl apiosylglucoside × $FeSO_4$ ($t_{0'}$); (**N**) chrysoeriol 7-*O*-6″-acetyl apiosylglucoside × $FeSO_4$ (t_{24}); (**O**) chrysoeriol 7-*O*-6″-malonyl apiosylglucoside × $FeSO_4$ ($t_{0'}$); and (**P**) chrysoeriol 7-*O*-6″-malonyl apiosylglucoside × $FeSO_4$ (t_{24}).

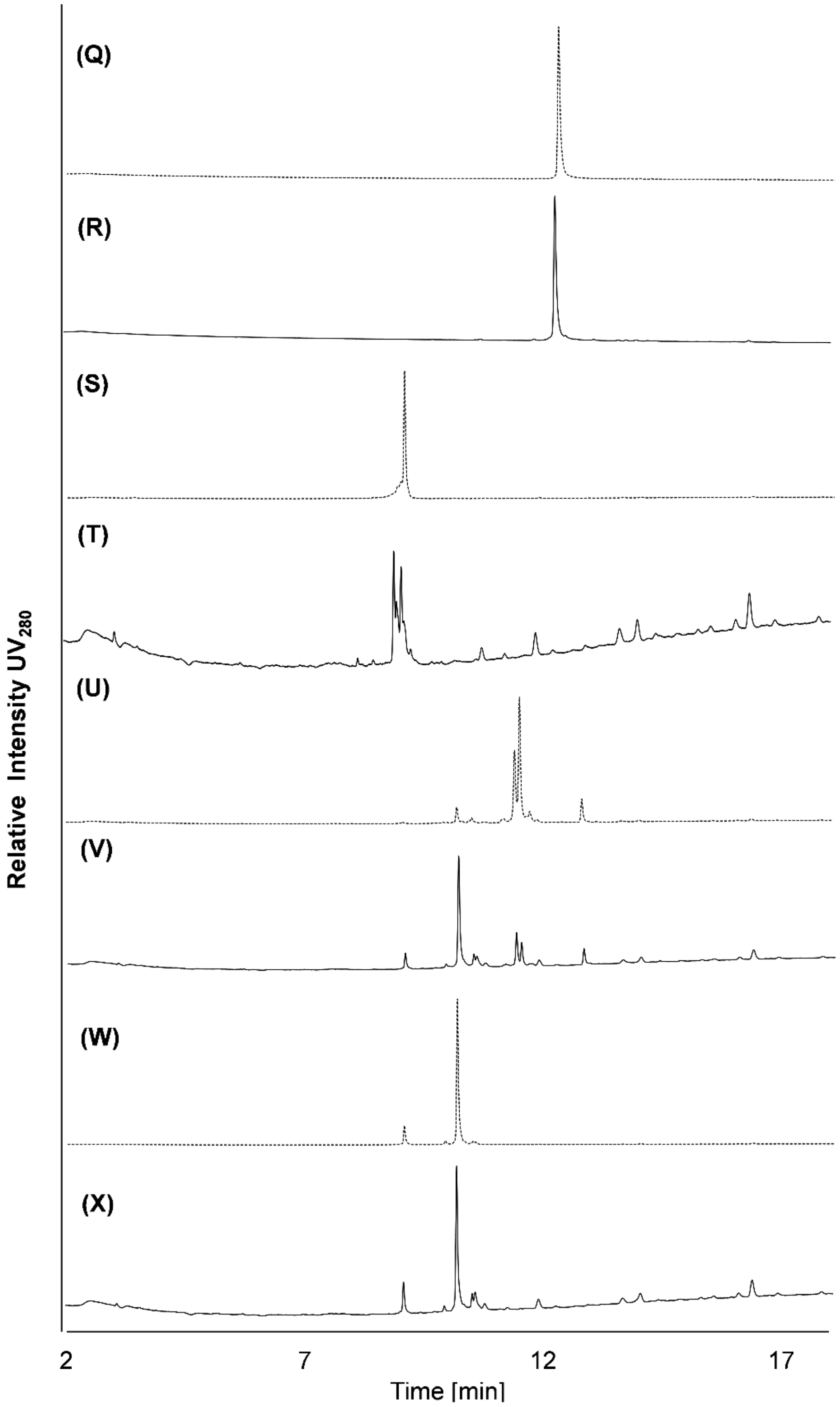

Figure SI-17 continued. Combined RP-UHPLC-PDA (280 nm) chromatograms from the WS and DS fractions of (**Q**) luteolin × $FeSO_4$ ($t_{0'}$); (**R**) luteolin × $FeSO_4$ (t_{24}); (**S**) luteolin 7-*O*-apiosylglucoside × $FeSO_4$ ($t_{0'}$); (**T**) luteolin 7-*O*-apiosylglucoside × $FeSO_4$ (t_{24}); (**U**) luteolin 7-*O*-6″-acetyl apiosylglucoside × $FeSO_4$ ($t_{0'}$); (**V**) luteolin 7-*O*-6″-acetyl apiosylglucoside × $FeSO_4$ (t_{24}); (**W**) luteolin 7-*O*-6″-malonyl apiosylglucoside × $FeSO_4$ ($t_{0'}$); and (**X**) luteolin 7-*O*-6″-malonyl apiosylglucoside × $FeSO_4$ (t_{24}).

Supporting Information with “Interactions of natural flavones with iron are affected by 7-*O*-glycosylation, but not by additional 6″-*O*-acylation” by Bijlsma, de Bruijn, Koppelaar, Sanders, Velikov, and Vincken.

We also investigated the stability of the malonyl, acetyl, and apiosylglucosyl substituents in presence of iron. For the malonyl apiosylglucosides, we investigated the effect of iron on demalonylation and decarboxylation by screening for formation of the corresponding apiosylglucosides and acetyl apiosylglucosides over time in presence and absence of iron (**Fig. SI-18a**). In all tested samples demalonylation (~ 14%) occurred more extensively than decarboxylation (~ 1 %) after 24 h incubation. For the acetyl apiosylglucosides, we investigated the effect of iron on deacetylation by screening for formation of the corresponding apiosylglucosides (**Fig. SI-18a**). The acetyl group was more stable than the malonyl group, as < 1 % of deacetylation was observed. No relationship between the presence of iron and demalonylation or deacetylation was observed. Moreover, no deglycosylation of the samples in absence or presence of iron was observed.

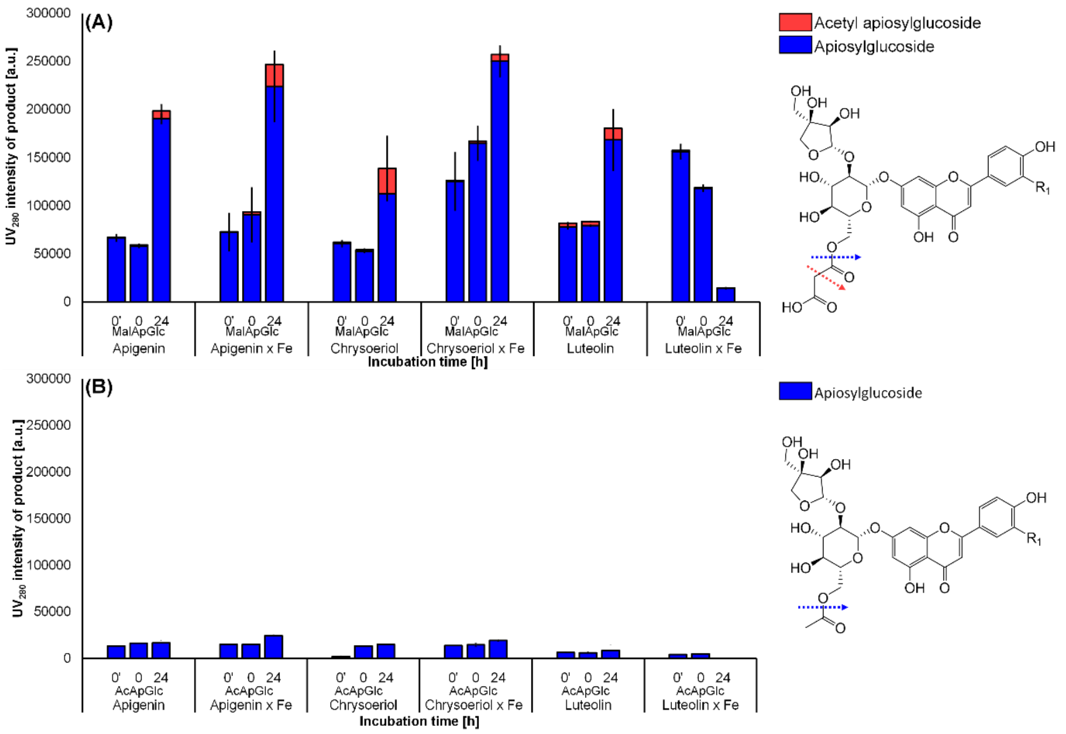

Fig. SI-18. Stability of the malonylated (**A**) and acetylated (**B**) flavone 7-*O*-apiosylglucosides in presence or absence of equimolar concentration $FeSO_4$ before adjustment of the pH ($t_{0'}$) and after 0 and 24 h of incubation at pH 6.5 in aqueous solution on product formation was investigated. Error bars indicate the standard deviation of independent duplicates.

References

(1) Narváez-Cuenca, C.-E.; Vincken, J.-P.; Gruppen, H. Identification and quantification of (dihydro) hydroxycinnamic acids and their conjugates in potato by UHPLC–DAD–ESI-MS^n. *Food Chem.* **2012**, *130* (3), 730-738. DOI: 10.1016/j.foodchem.2011.04.050.

(2) Plazonić, A.; Bucar, F.; Maleš, Ž.; Mornar, A.; Nigović, B.; Kujundžić, N. Identification and quantification of flavonoids and phenolic acids in burr parsley (*Caucalis platycarpos* L.), using high-performance liquid chromatography with diode array detection and electrospray ionization mass spectrometry. *Molecules* **2009**, *14* (7), 2466-2490. DOI: 10.3390/molecules14072466.

(3) Lin, L.-Z.; Harnly, J. M. A screening method for the identification of glycosylated flavonoids and other phenolic compounds using a standard analytical approach for all plant materials. *J. Agric. Food Chem.* **2007**, *55* (4), 1084-1096. DOI: 10.1021/jf062431s.

(4) Fernández-Poyatos, M. D. P.; Ruiz-Medina, A.; Zengin, G.; Llorent-Martínez, E. J. Phenolic characterization, antioxidant activity, and enzyme inhibitory properties of *Berberis thunbergii* DC. Leaves: A valuable source of phenolic acids. *Molecules* **2019**, *24* (22). DOI: 10.3390/molecules24224171.

(5) Lin, L.-Z.; Lu, S.; Harnly, J. M. Detection and quantification of glycosylated flavonoid malonates in celery, chinese celery, and celery seed by LC-DAD-ESI/MS. *J. Agric. Food Chem.* **2007**, *55* (4), 1321-1326. DOI: 10.1021/jf0624796.

(6) Cavaliere, C.; Foglia, P.; Pastorini, E.; Samperi, R.; Laganà, A. Identification and mass spectrometric characterization of glycosylated flavonoids in *Triticum durum* plants by high-performance liquid chromatography with tandem mass spectrometry. *Rapid Commun. Mass Spectrom.* **2005**, *19* (21), 3143-3158. DOI: 10.1002/rcm.2185.

(7) Hostetler, G. L.; Riedl, K. M.; Schwartz, S. J. Endogenous enzymes, heat, and pH affect flavone profiles in parsley (*Petroselinum crispum* var. *neapolitanum*) and celery (*Apium graveolens*) during juice processing. *J. Agric. Food Chem.* **2012**, *60* (1), 202-208. DOI: 10.1021/jf2033736.

(8) Munekata, P. E. S.; Alcántara, C.; Žugčić, T.; Abdelkebir, R.; Collado, M. C.; García-Pérez, J. V.; Jambrak, A. R.; Gavahian, M.; Barba, F. J.; Lorenzo, J. M. Impact of ultrasound-assisted extraction and solvent composition on bioactive compounds and in vitro biological activities of thyme and rosemary. *Food Res. Int.* **2020**, *134*, 109242. DOI: 10.1016/j.foodres.2020.109242.

(9) Ma, Y.; Kosińska-Cagnazzo, A.; Kerr, W. L.; Amarowicz, R.; Swanson, R. B.; Pegg, R. B. Separation and characterization of phenolic compounds from dry-blanched peanut skins by liquid chromatography–electrospray ionization mass spectrometry. *J. Chromatogr. A* **2014**, *1356*, 64-81.

(10) Bresciani, L.; Favari, C.; Calani, L.; Francinelli, V.; Riva, A.; Petrangolini, G.; Allegrini, P.; Mena, P.; Del Rio, D. The effect of formulation of curcuminoids on their metabolism by human colonic microbiota. *Molecules* **2020**, *25* (4), 940. DOI: 10.3390/molecules25040940.

(11) Eckey-Kaltenbach, H.; Heller, W.; Sonnenbichler, J.; Zetl, I.; Schäfer, W.; Ernst, D.; Sandermann, H. Oxidative stress and plant secondary metabolism: 6″-O-malonylapiin in parsley. *Phytochemistry* **1993**, *34* (3), 687-691. DOI: 10.1016/0031-9422(93)85340-W.

(12) Yoshikawa, M.; Uemura, T.; Shimoda, H.; Kishi, A.; Kawahara, Y.; Matsuda, H. Medicinal foodstuffs. XVIII. Phytoestrogens from the aerial part of *Petroselinum crispum* Mill. (parsley) and structures of 6"-acetylapiin and a new monoterpene glycoside, petroside. *Chem. Pharm. Bull.* **2000**, *48* (7), 1039-1044. DOI: 10.1248/cpb.48.1039.

(13) Park, Y.; Moon, B. H.; Yang, H.; Lee, Y.; Lee, E.; Lim, Y. Complete assignments of NMR data of 13 hydroxymethoxyflavones. *Magn. Reson. Chem.* **2007**, *45* (12), 1072-1075. DOI: 10.1002/mrc.2063.

(14) Momin, R. A.; Nair, M. G. Antioxidant, cyclooxygenase and topoisomerase inhibitory compounds from *Apium graveolens* Linn. seeds. *Phytomedicine* **2002**, *9* (4), 312-318. DOI: 10.1078/0944-7113-00131.

(15) Boulton, R. The copigmentation of anthocyanins and its role in the color of red wine: A critical review. *Am. J. Enol. Vitic.* **2001**, *52* (2), 67-87.
